# Supplementary material for: Observation of Antichiral Hinge States in a Three-dimensional Gyromagnetic Photonic Crystal
Source: Nat Commun. 2026 Aug 26;17:10186. doi: 10.1038/s41467-026-77286-6 (PMC13612583; doi:10.1038/s41467-026-77286-6)
Supplement: Supplementary file 1 [file 41467_2026_77286_MOESM1_ESM.pdf]

Supplemental Material for
"Observation of Antichiral Hinge States in a Three-dimensional Gyromagnetic Photonic Crystal"

Ziyao Wang,[1,*], Tianzhi Xia,[2,*,†], Han-Rong Xia[1,‡], and Zhen Gao[1,§]
[1]State Key Laboratory of Optical Fiber and Cable Manufacturing Technology, Department of Electronic and Electrical Engineering, Guangdong Key Laboratory of Integrated Optoelectronics Intellisense, Southern University of Science and Technology, Shenzhen 518055, China.
[2]College of Physics and Electronic Engineering, Qujing Normal University, Qujing 655011, China
*These authors contributed equally to this work.
Corresponding Email: xiatianzhi@mail.qjnu.edu.cn, xiahr@sustech.edu.cn, gaoz@sustech.edu.cn

This file includes:

1. Hamiltonian of the Theoretical Model

2. Calculation of Isofrequency Contours

3. Formation Mechanisms of the Nodal Structures

4. Detailed Projected Dispersion with Small and Large Interlayer Couplings

5. Calculation of Nested Wilson Loop and Surface Polarization

6. Theoretical Model with Larger Interlayer Couplings

7. Eigenfield Profiles of Antichiral Surface States

8. Experimental Results of the Two Bottom Hinges

9. Experimental Setup

10. Robustness of the Antichiral Hinge States against Local Structural Defects

11. Photonic Device Applications Based on Antichiral Hinge States.

1. Hamiltonian of the Theoretical Model

We consider a three-dimensional (3D) tight-binding model constructed by stacking a two-dimensional (2D) modified Haldane model (MHM) with dimerized interlayer couplings. The full Hamiltonian in momentum space takes the block-diagonal form:

$$H = H_{xy} \otimes \tau_0 + \sigma_0 \otimes H_z, \tag{S1}$$

where σ and τ denote Pauli matrices acting on the sublattice (A, B) and layer degrees of freedom, respectively. The in-plane Hamiltonian H_{xy} describes an MHM where particle-hole symmetry is explicitly broken by sublattice-independent next-nearest-neighbor (NNN) hoppings. We decompose the in-plane Hamiltonian as

$$H_{xy}(\boldsymbol{k}_{||}) = d_0(\boldsymbol{k}_{||})\sigma_0 + d_1(\boldsymbol{k}_{||})\sigma_1 + d_2(\boldsymbol{k}_{||})\sigma_2 + d_3(\boldsymbol{k}_{||})\sigma_3, \tag{S2}$$

with coefficients given by: $d_0 = 2t_2 \sum_{n=1}^{3} \cos(\phi - \boldsymbol{k}_{||} \cdot \boldsymbol{b}_n)$, $d_1 = 2t_1 \sum_{n=1}^{3} \cos(\boldsymbol{k}_{||} \cdot \boldsymbol{a}_n)$, $d_2 = 2t_1 \sum_{n=1}^{3} \sin(\boldsymbol{k}_{||} \cdot \boldsymbol{a}_n)$, and $d_3 = M$. Here, $\boldsymbol{a}_n$ is the nearest-neighbor position vector, and $\boldsymbol{b}_n$ represents the NNN position vector. Crucially, the $\boldsymbol{k}$-dependent $d_0\sigma_0$ term introduces a tilt in the energy dispersion, which is a prerequisite for the emergence of antichiral edge states. The out-of-plane Hamiltonian $H_z(\boldsymbol{k}_z)$ introduces an Su-Schrieffer-Heeger (SSH) dimerization of interlayer couplings. In the basis of the two-layer unit cell, it reads $H_z(\boldsymbol{k}_z) = h_1(\boldsymbol{k}_z)\tau_1 + h_2(\boldsymbol{k}_z)\tau_2$, with $h_1(\boldsymbol{k}_z) = t_3 + t_4\cos(\boldsymbol{k}_z h_z)$ and $h_2(\boldsymbol{k}_z) = t_4\sin(\boldsymbol{k}_z h_z)$.

2. Calculation of Isofrequency Contours

To verify the 3D nodal structures and avoid FFT resolution limits in finite-size 3D simulations, we employ the tight-binding Green's function method to calculate the momentum-resolved local density of states (LDOS). This approach generates the exact momentum-space spectral function $A(k, \mathrm{E})$, which serves as the theoretical equivalent of the isofrequency contours reconstructed from the measured near-field distributions. To map the LDOS on different projection planes, we construct finite-thickness slab models along three lattice directions. For instance, to investigate the spectral properties projected onto the k_x-k_y plane, we construct an N_z-layer slab model $H_{slab}(k_{\parallel})$ by opening the boundary along the *z*-direction while maintaining periodicity in the *x*-*y* plane. The retarded Green's function of the slab is defined as:

$$\mathrm{G}_{nn}(k_{\parallel}, \mathrm{E}) = \frac{1}{[(\mathrm{E} + \mathrm{i}\eta)\mathrm{I} - H_{slab}(k_{\parallel})]} \tag{S3}$$

Where E is the targeted excitation energy, I is the identity matrix, and η is the intrinsic loss. The momentum-space LDOS for a specific layer *n* can then be extracted from the imaginary part of the partial trace of the Green's function:

$$A_n(k_{\parallel}, \mathrm{E}) = -\frac{1}{\pi} Im\left[\mathrm{Tr}\left(\mathrm{G}_{nn}(k_{\parallel}, \mathrm{E})\right)\right] \quad \text{(S4)}$$

By extracting the diagonal blocks corresponding to the bulk middle layer and the top surface layer, we can separately visualize the isofrequency contour projected onto the bulk and the surface.

Supplementary Fig. 1 presents the theoretically calculated momentum-space LDOS at the nodal energy E = -0.24. At this specific energy, only the nodal structures around the *K-H* path (as depicted in Fig. 2c) are excited. As shown in the k_x-k_y projections (Supplementary Fig. 1a and Supplementary Fig. 1d), the white dashed hexagon delineates the boundary of the first Brillouin zone, around whose three vertices the nodal structure clearly emerges. Specifically, the nodal line projects onto the k_x-k_y plane as distinct singular points at these vertices, while the nodal surface manifests as bright continuous rings surrounding them. The geometric profile perfectly corroborates the 3D nodal structure illustrated in Fig. 2d.

In the k_x-k_z cross-sections (Supplementary Fig. 1b and Supplementary Fig. 1e), we indicate the positions of the nodal surfaces using dashed white lines. It is evident that the bulk spectra exhibit continuous vertical bright walls extending along the entire k_z direction around $k_x = 2\pi/3$. Furthermore, the antichiral surface states are prominently bright at this energy in Supplementary Fig. 1e, which is in excellent agreement with the projected band dispersion depicted in Fig. 2e.

Finally, the k_y-k_z projections illustrated in Supplementary Fig. 1c and Supplementary Fig. 1f further confirm the phenomenon that the nodal structure extends continuously along the whole k_z direction around $k_y = 0$, which is consistent with Fig. 2d. Similarly, the corresponding isofrequency contour at E = 0.24 is presented in Supplementary Fig. 2, exhibiting similar topological features.

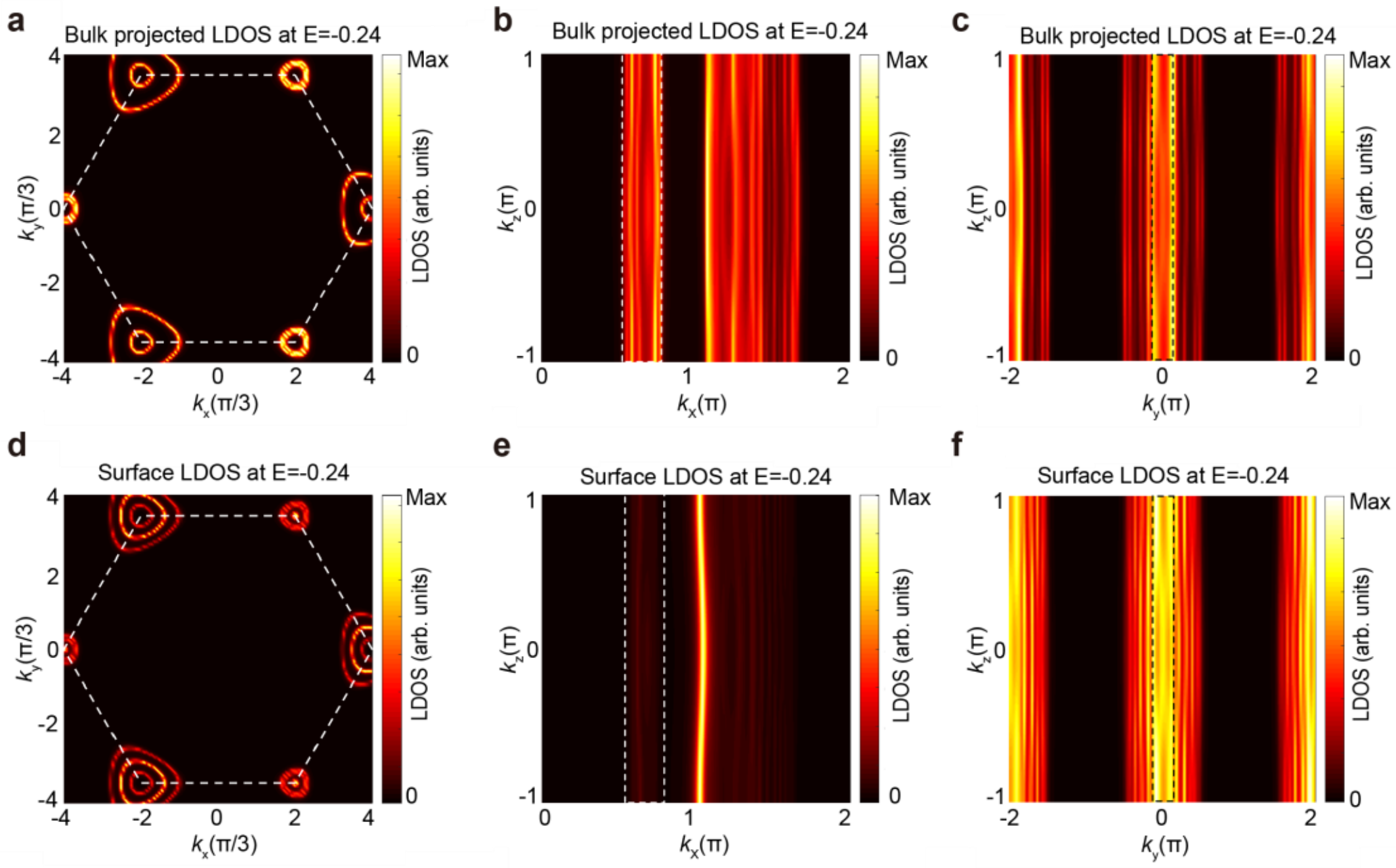

Supplementary Fig. 1 | Theoretically calculated momentum-space LDOS at the nodal frequency E = -0.24. a-c Bulk isofrequency contours projected onto the k_x-k_y, k_x-k_z and k_y-k_z planes, respectively. **d-f** Surface isofrequency contours on the k_x-k_y, k_x-k_z and k_y-k_z planes, respectively.

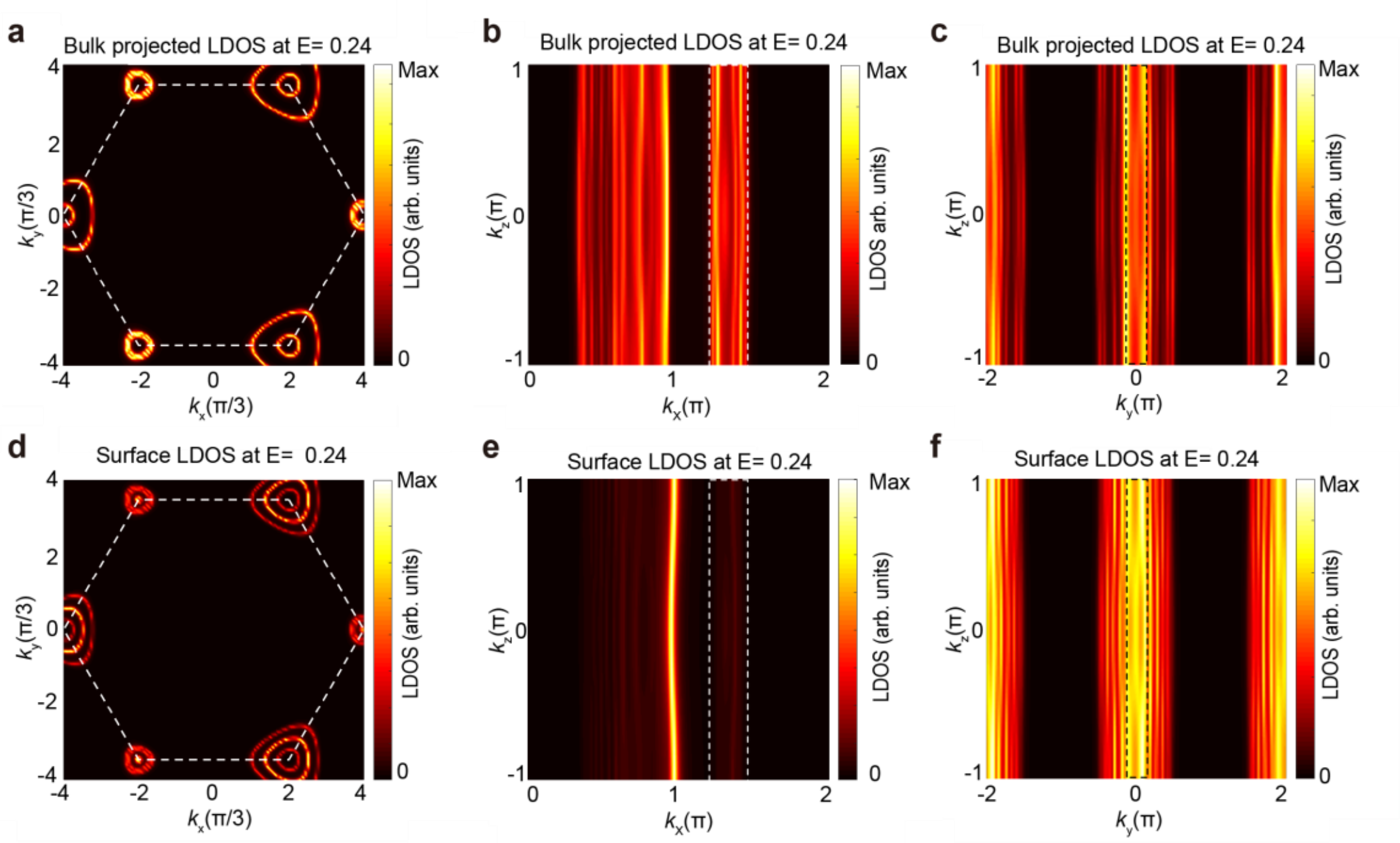

Supplementary Fig. 2 | Theoretically calculated momentum-space LDOS at the nodal frequency E = 0.24. a-c Bulk isofrequency contours projected onto the k_x-k_y, k_x-k_z and k_y-k_z planes, respectively. **d-f** Surface isofrequency contours on the k_x-k_y, k_x-k_z and k_y-k_z planes, respectively.

3. Formation Mechanisms of the Nodal Structures

To elucidate the fundamental origin of the coexisting nodal lines (NLs) and nodal surfaces (NSs) in our theoretical model, we analytically derive the band degeneracies from the total Hamiltonian. As depicted in the main text, the eigenvalues of the total Hamiltonian are given by $E = E_{xy} + E_z$. By decomposing the in-plane MHM into Pauli matrices, we can obtain the in-plane Hamiltonian described in Eq. (S2). In the topological semimetal phase discussed in the main text, the sublattice mass term is set to be zero ($d_3 = M = 0$). Therefore, the in-plane eigenvalues are given by:

$$E_{xy}(\boldsymbol{k}_{||}) = d_0(\boldsymbol{k}_{||}) \pm \sqrt{d_1(\boldsymbol{k}_{||})^2 + d_2(\boldsymbol{k}_{||})^2}, \qquad \text{(S5)}$$

The out-of-plane SSH Hamiltonian is defined as $H_z(\boldsymbol{k}_z) = h_1(\boldsymbol{k}_z)\tau_1 + h_2(\boldsymbol{k}_z)\tau_2$. Its eigenvalues are strictly symmetric around zero:

$$E_z(\boldsymbol{k}_z) = \pm\sqrt{h_1(\boldsymbol{k}_z)^2 + h_2(\boldsymbol{k}_z)^2}, \qquad \text{(S6)}$$

Combining these, the four bulk bands of the 3D tight-binding model can be described by the dispersion relation:

$$E(\boldsymbol{k}_{||}, \boldsymbol{k}_z) = d_0(\boldsymbol{k}_{||}) \pm \sqrt{d_1(\boldsymbol{k}_{||})^2 + d_2(\boldsymbol{k}_{||})^2} \pm \sqrt{h_1(\boldsymbol{k}_z)^2 + h_2(\boldsymbol{k}_z)^2}, \quad \text{(S7)}$$

To simplify the analysis of gap closings, we define the in-plane Dirac dispersion term as $\rho(\boldsymbol{k}_{||}) = \sqrt{d_1(\boldsymbol{k}_{||})^2 + d_2(\boldsymbol{k}_{||})^2}$ and the out-of-plane dimerization term as $\Delta(\boldsymbol{k}_z) = \sqrt{h_1(\boldsymbol{k}_z)^2 + h_2(\boldsymbol{k}_z)^2}$.

Nodal lines arise from the degeneracies between the first and second bands, as well as between the third and fourth bands. This occurs when the in-plane gap closes, meaning $\rho(\boldsymbol{k}_{||}) = 0$. Due to the in-plane C_3 symmetry, this condition is satisfied exclusively at the high-symmetry points K and K' in the 2D Brillouin zone, where $d_1 = d_2 = 0$. Under this condition, the energy eigenvalues reduce to $E = d_0(k_{K/K'}) \pm \Delta(\boldsymbol{k}_z)$. Because this twofold degeneracy holds for any value of $\boldsymbol{k}_z$, the degenerate points extend straightly along the $\boldsymbol{k}_z$ direction, forming the NLs.

The NS corresponds to the crossing between the second and third bands. This specific degeneracy requires the upper branch of the lower doublet and the lower branch of the upper doublet to intersect. Mathematically, this crossing occurs when $\rho(\boldsymbol{k}_{||}) = \Delta(\boldsymbol{k}_z)$, or equivalently:

$$\sqrt{d_1(\boldsymbol{k}_{||})^2 + d_2(\boldsymbol{k}_{||})^2} = \sqrt{h_1(\boldsymbol{k}_z)^2 + h_2(\boldsymbol{k}_z)^2}, \quad \text{(S8)}$$

This equation defines a continuous 2D manifold (surface) in the 3D momentum space. Because $\rho(\boldsymbol{k}_{||})$ increases linearly as one moves away from the Dirac points (K and K') in the $\boldsymbol{k}_{||}$ plane, and $\Delta(\boldsymbol{k}_z)$ acts as a $\boldsymbol{k}_z$-dependent radius, the condition $\rho(\boldsymbol{k}_{||}) = \Delta(\boldsymbol{k}_z)$ naturally forms closed, tube-like surfaces.

Consequently, these NSs inherently wrap around the NLs at K and K'. This mathematical demonstration shows how the NS-wrapping-NL configuration arises from the interplay between the in-plane Dirac dispersion and the out-of-plane nodal limitations imposed by the dimerized interlayer couplings.

4. Detailed Projected Dispersion with Small and Large Interlayer Couplings

In the calculated hinge dispersion, in addition to the antichiral hinge and surface states, the spectrum inherently contains surface states (blue dots) localized at the top and bottom surfaces. Based on the separable Hamiltonian $H = H_{xy} \otimes \tau_0 + \sigma_0 \otimes H_z$, these top and bottom surface states emerge from the hybridization of the in-plane bulk modes and the out-of-plane SSH topological end

modes. As a result, the dispersion of these surface states largely mirrors the continuous energy bands of the in-plane bulk states E_{xy}, while their spatial energy distributions are confined to the upper and lower facets of the finite 3D tight-binding model. In our numerical calculations, we evaluate the spatial energy distribution of each eigenmode within the 3D supercell. We partition the lattice sites into four regions: the bulk interior, the top and bottom facets, the side facets, and the 1D hinges at the intersections of adjacent side facets. By integrating the field intensity over each region, we classify each mode as a bulk state, a top/bottom surface state, an antichiral side-surface state, or a hinge state according to the region where its energy is predominantly localized. We present detailed projected dispersions for the parameters used in the main text (Supplementary Fig. 3a) and for a case with stronger interlayer coupling along the z-direction (Supplementary Fig. 3b), corresponding to Supplementary Fig. 6.

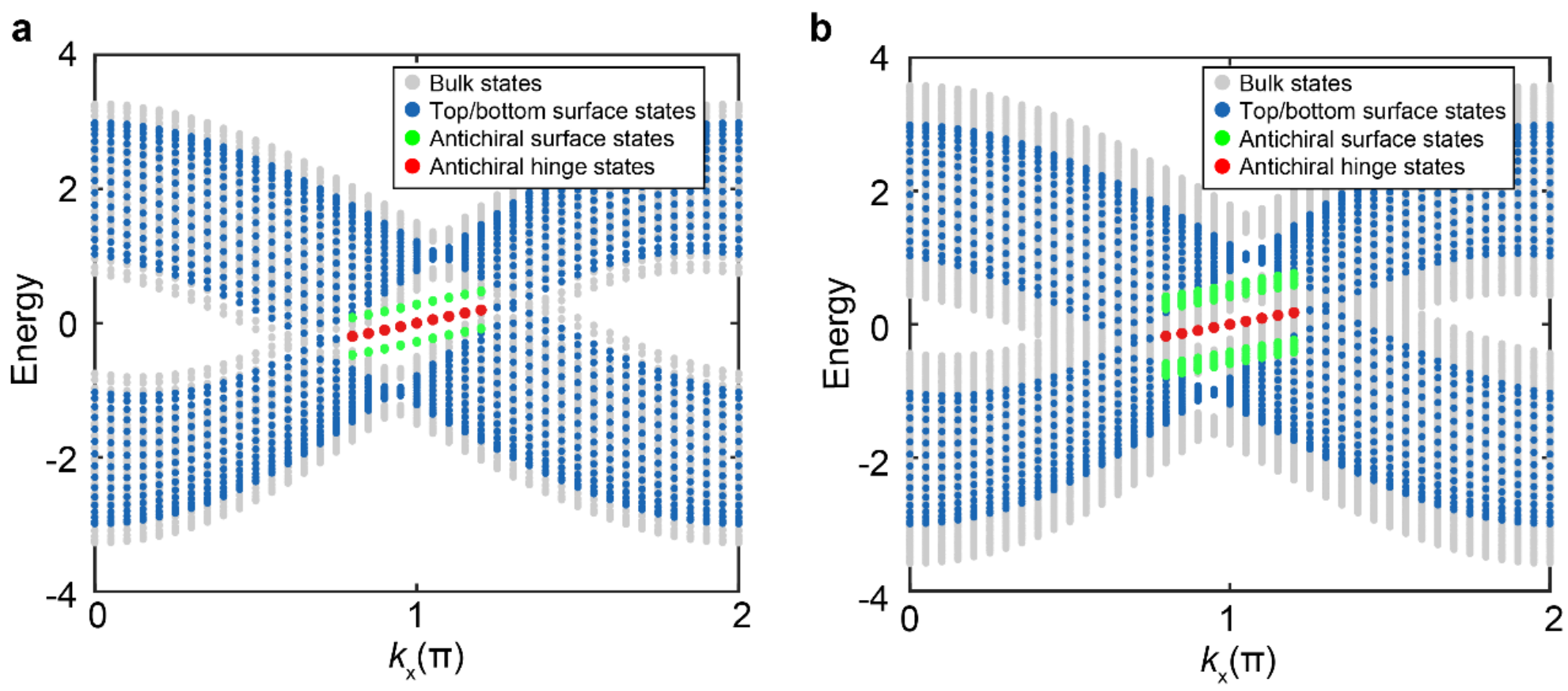

Supplementary Fig. 3 | Detailed projected dispersion along the k_x direction with small and larger interlayer couplings. a Dispersion computed with small interlayer couplings ($M = 0$, $t_1 = 1$, $t_2 = 1/18$, $\varphi = \pi/2$, $t_3 = 0.2t_2$, and $t_4 = 5t_2$). **b** Dispersion with larger interlayer couplings ($t_3 = 0.1$, $t_4 = 0.5$), corresponding to Supplementary Fig. 6. The red and green circles highlight the antichiral hinge and surface states, respectively. The gray and blue dots indicate the bulk and top- and bottom-surface states.

5. Calculation of Nested Wilson Loop and Surface Polarization

To characterize the higher-order bulk band topology of the antichiral hinge states, we implement the nested Wilson loop method to calculate the nested polarization $p_y^{v_z}$ based on the 3D tight-binding Hamiltonian.

For a half-filled system with N_{occ} occupied bands (here N_{occ}=2), we first construct the Wilson loop operator $\mathcal{W}_z(k_x, k_y)$ along the k_z direction by taking the path-ordered product of the overlap matrices over a period, defined as $[F_{z,k}]_{mn}=\langle u_{m,k} | u_{n,k+\Delta k_z}\rangle$, where $|u_{m,k}\rangle$ represents the occupied Bloch wavefunctions.

Diagonalizing this out-of-plane Wilson loop operator yields the k_z-directed Wannier centers $v_z^j(k_x, k_y)$ and the corresponding Wannier eigenstates $|\omega_{z,j}(k_x, k_y)\rangle$ via the $\mathcal{W}_z|\omega_{z,j}\rangle = e^{i2\pi v_z^j}|\omega_{z,j}\rangle$. As illustrated in Supplementary Fig. 4a, the Wannier bands do not cross the entire Brillouin zone due to dimerized interlayer couplings along the *z*-axis. Specifically, the Wannier centers are tightly localized near $v_z = 0$ in momentum regions away from the projected nodal surfaces (NSs), whereas they shift toward ± 0.5 in the vicinity of the NSs. Cross-sectional profiles of the Wannier bands at selected transverse momenta (Supplementary Figs. 4b-4f) further confirm the strict absence of band crossings. This gap cleanly splits the Wannier bands into separate sectors. We then isolate the upper Wannier sector by extracting the specific eigenvector matrix $V_w(k_x, k_y)$ corresponding to the upper branch to serve as our localized Wannier basis.

Subsequently, the Bloch state overlap matrices along the k_y direction, $[F_{y,k}]_{mn}=\left\langle u_{m,k} \middle| u_{n,k+\Delta k_y}\right\rangle$, are calculated across the momentum space. To maintain strict numerical unitarity on the discretized grid and ensure gauge smoothness during propagation, a singular value decomposition is performed on $F_{y,k}$ before projecting it onto the Wannier basis, which systematically constructs the nested overlap matrix $\tilde{F}_{y,k} = V_w{}^{\dagger} F_{y,k} V_w(k + \Delta k_y)$. Finally, the nested Wilson loop operator $\widetilde{\mathcal{W}}_y(k_x)$ is obtained by the product of these projected matrices along the closed k_y loop, $\widetilde{\mathcal{W}}_y(k_x) = \prod_{k_y} \tilde{F}_{y,k}$. Because the occupied subspace has been successfully projected into a single Wannier sector, $\widetilde{\mathcal{W}}_y(k_x)$ reduces to a scalar complex number whose phase directly yields the quantized nested polarization $p_y^{v_z} = \frac{1}{2\pi}\mathrm{Im}[\ln \widetilde{\mathcal{W}}_y(k_x)]$ mod 1.

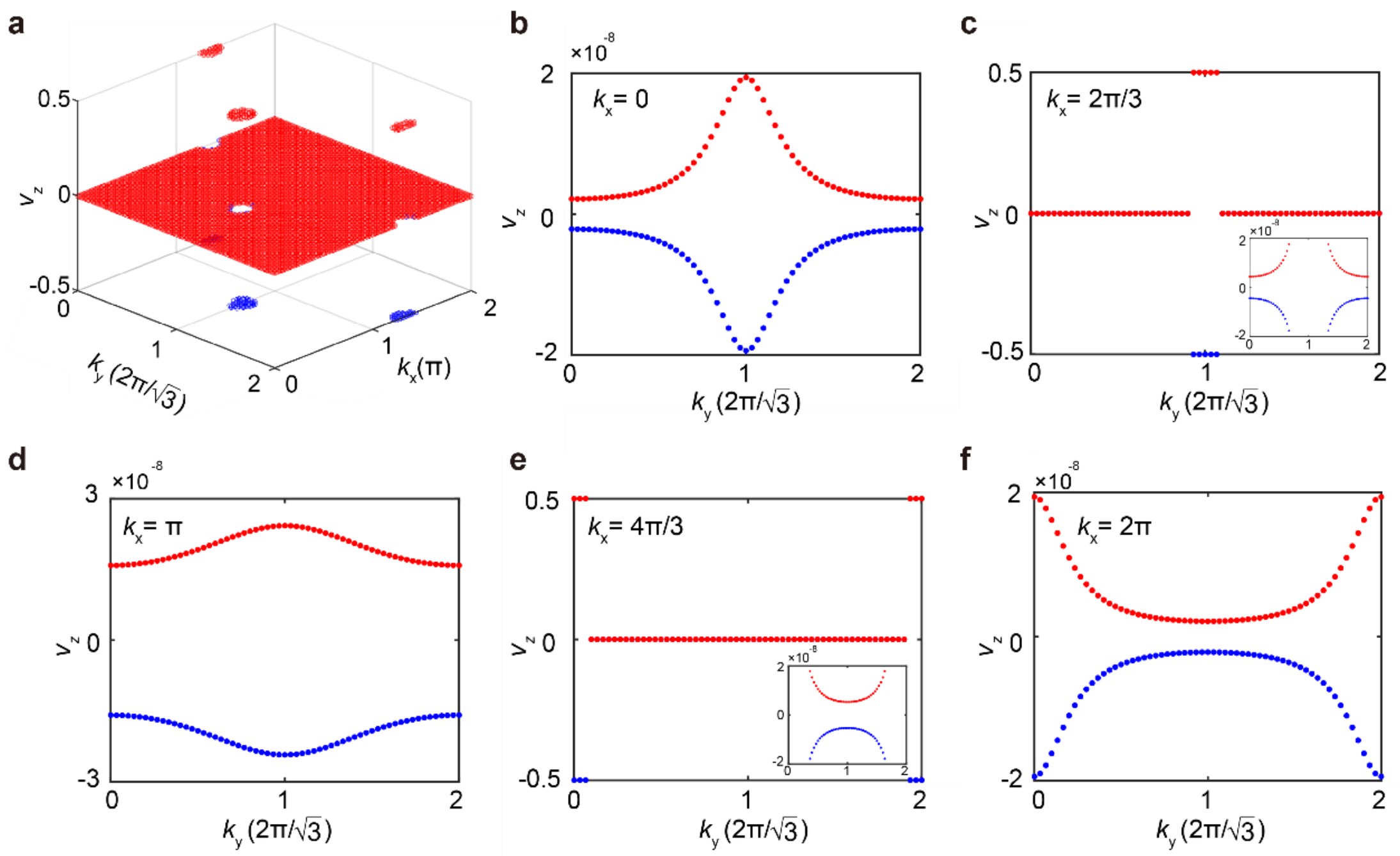

Supplementary Fig. 4 | Evolution of Wannier centers and the global Wannier band gap. a Three-dimensional distribution of the Wannier centers v_z over the two-dimensional momentum space (k_x, k_y) of the first Brillouin zone. The red and blue markers distinguish the two constituent Wannier bands. **b-f** Cross-sectional profiles of the Wannier bands v_z along the k_y direction at selected transverse momenta: (b) $k_x = 0$, (c) $k_x = \frac{2\pi}{3}$, (d) $k_x = \pi$, (e) $k_x = \frac{4\pi}{3}$, (f) $k_x = 2\pi$. The insets in (c) and (e) provide magnified views of the transition regions, confirming the absence of band crossings. The two Wannier bands remain separated by a finite gap across the entire Brillouin zone, ensuring that the projection onto a single Wannier sector for the subsequent nested Wilson loop calculation is topologically well-defined.

To explicitly confirm that the antichiral hinge states originate from surface-obstructed topology, we evaluate the surface Wannier polarization $v_z^{\text{surface}}(k_x)$ for the *y*-normal surfaces. While the nested Wilson loop captures the global 3D bulk properties, our antichiral hinge states emerge directly from the gapped 2D surface spectrum. By isolating the antichiral surface band and tracking its evolution along k_z, we compute the one-dimensional Zak phase:

$$v_z^{\text{surface}}(k_x) = \frac{1}{2\pi} \oint i \langle \psi^{\text{surface}}(k_x, k_z) | \partial_{k_z} | \psi^{\text{surface}}(k_x, k_z) \rangle \, dk_z \pmod{1} \qquad \text{(S9)}$$

As shown in Supplementary Fig. 5, the numerical results reveal v_z^{surface} that is strictly

quantized to 0.5 exclusively within the momentum interval 2/3 < k_x(π) < 4/3. The nontrivial quantization within this specific domain provides mathematical evidence that the 1D antichiral hinge channels are a manifestation of boundary-obstructed topology.

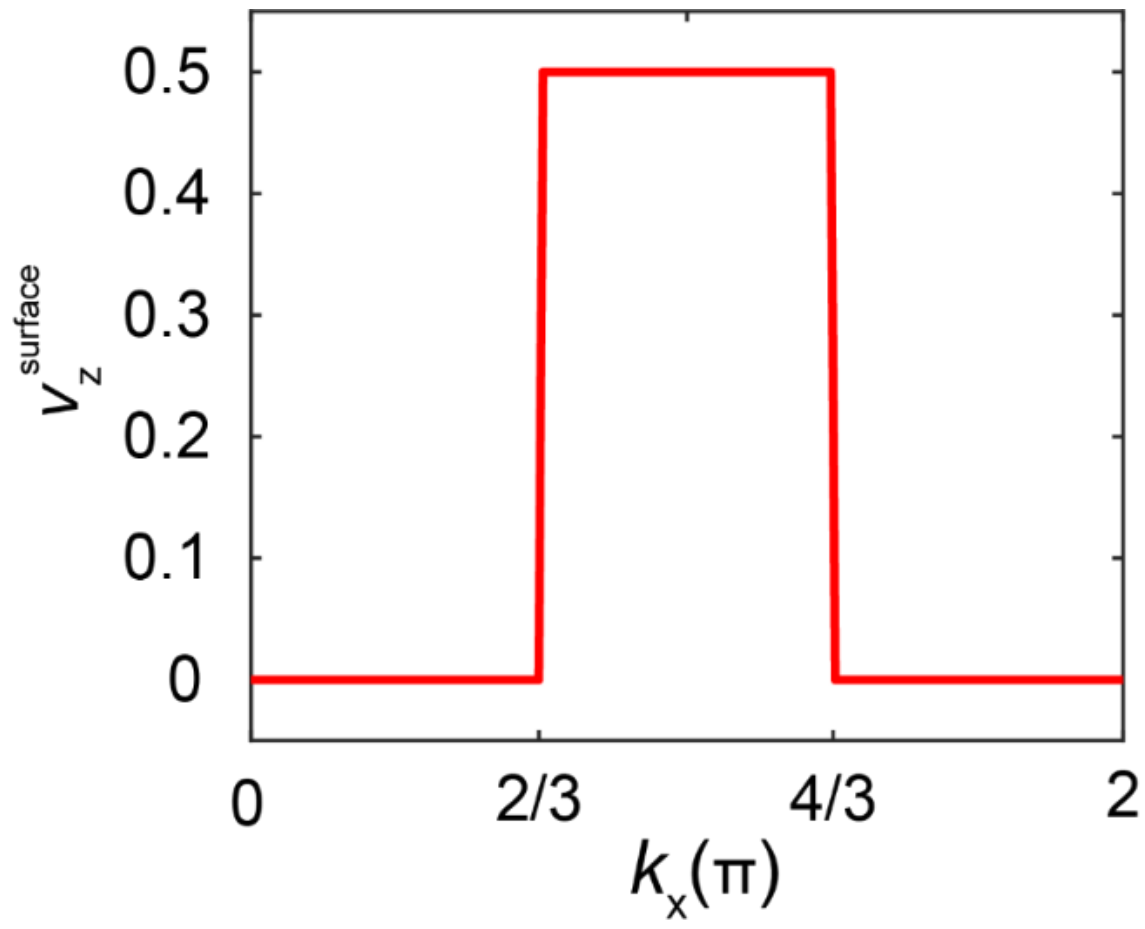

Supplementary Fig. 5 | Surface Wannier polarization of the *y*-normal surface. Calculated surface polarization v_z^{surface} as a function of the wavevector k_x.

6. Theoretical Model with Larger Interlayer Couplings

In the main text, we chose relatively weak interlayer couplings to facilitate the practical design and fabrication of the experimental sample. This choice of parameter, however, does not alter the underlying topological phase. To demonstrate this explicitly, we calculated the band structures for stronger interlayer couplings. As shown in Supplementary Fig. 6a, the system retains the same topological characteristics, including the robust coexistence of nodal-surface (NS) and nodal-line (NL) semimetal phases. Owing to the enhanced interlayer couplings, the bands along the *K–H, K′–H′*, and *Γ–A* directions acquire pronounced tilted dispersions. The corresponding NSs and NLs in 3D momentum space are visualized in Supplementary Fig. 6b. The projected band structure in Supplementary Fig. 6c further shows tilted, fourfold-degenerate antichiral hinge states that connect the projected NSs. Finally, as shown in Supplementary Fig. 6d, the nested Wilson-loop calculation for the full 3D Hamiltonian yields a quantized nested polarization, $p_y^{(v_z)} = 1/2$, throughout the momentum interval $k_{s1} < k_x < k_{s2}$ defined by the projected NSs.

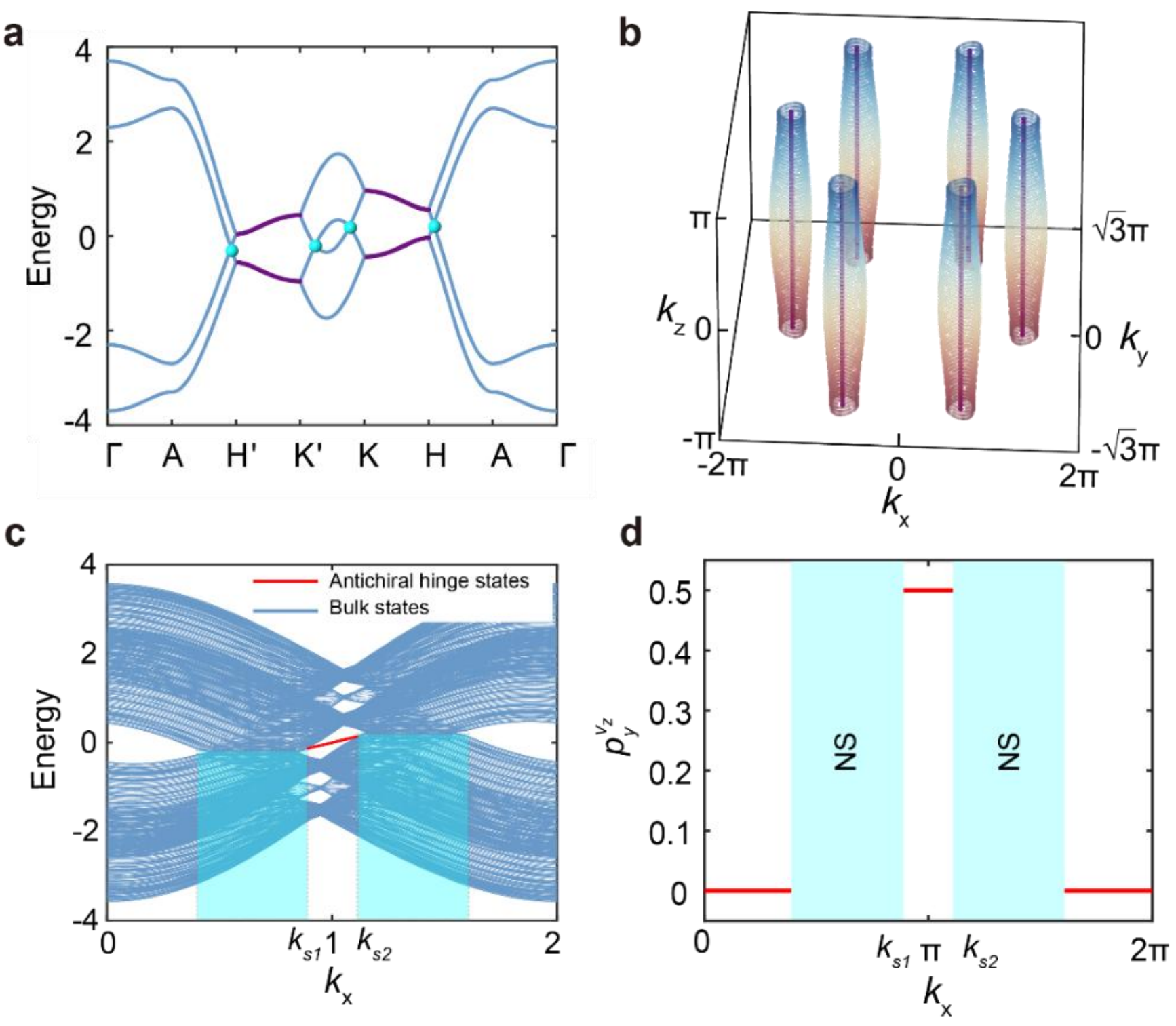

Supplementary Fig. 6 | Theoretical model with larger interlayer couplings. a Calculated bulk band structure of the 3D tight-binding model with parameters M = 0, t_1 = 1, t_2 = 0.05, t_3 = 0.1, t_4 = 0.5, $\varphi = \frac{\pi}{2}$. **b** Visualization of the nodal surfaces (colored surfaces) and nodal lines (purple lines) in the 3D momentum space. **c** Projected band dispersions of a 3D modified Haldane model with dimerized interlayer coupling that is finite in the *y*- and *z*-directions and periodic in the *x*-direction. **d** Nested polarization calculated from the full 3D Hamiltonian.

7. Eigenfield Profiles of Antichiral Surface States

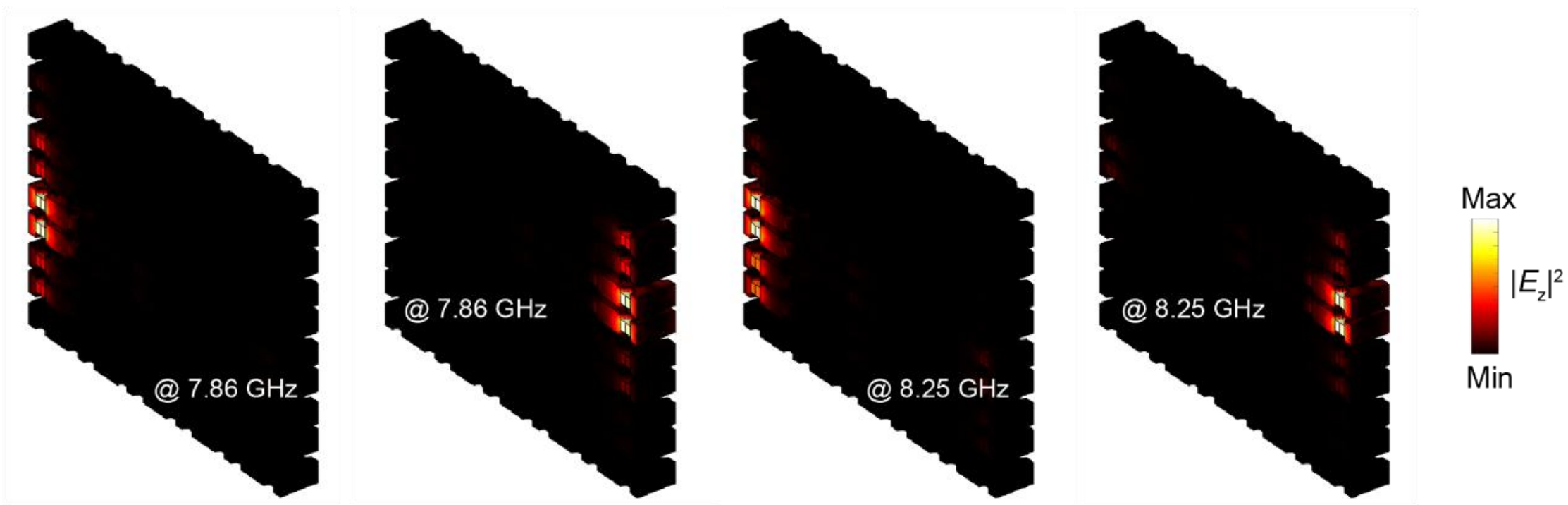

Supplementary Fig. 7 | Simulated electric field distributions of the antichiral surface states at $\boldsymbol{k_x = \pi/a}$.

8. Experimental Results of Two Bottom Hinges

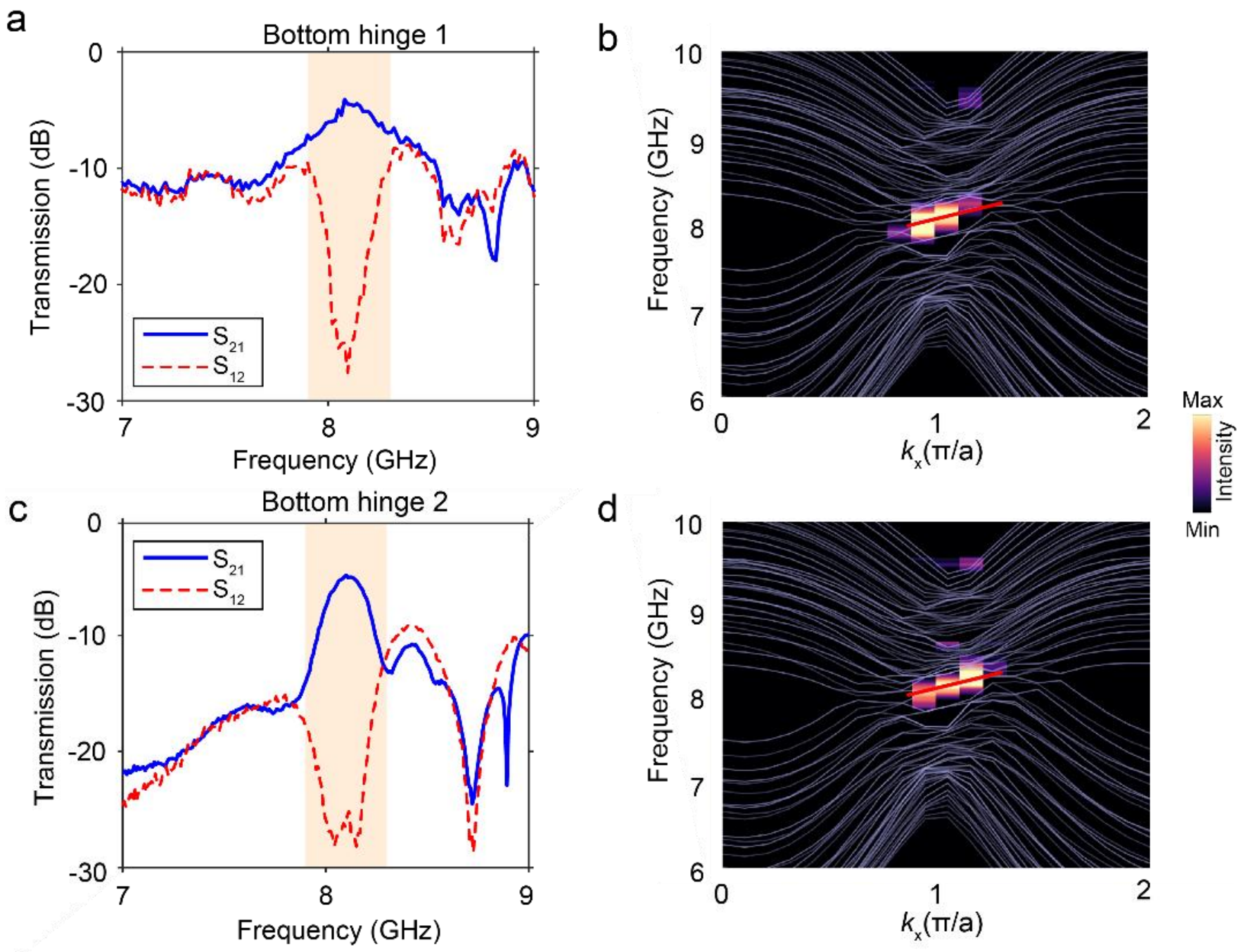

Supplementary Fig. 8 | Measured transmission spectra and dispersions of antichiral hinge states at two bottom hinges. a, **c** Measured forward (S_{21}) and backward (S_{12}) transmission spectra of the antichiral hinge states at the two bottom hinges. **b**, **d** Measured (colormaps) and simulated (red lines) dispersions of the antichiral hinge states at the two bottom hinges along the k_x direction.

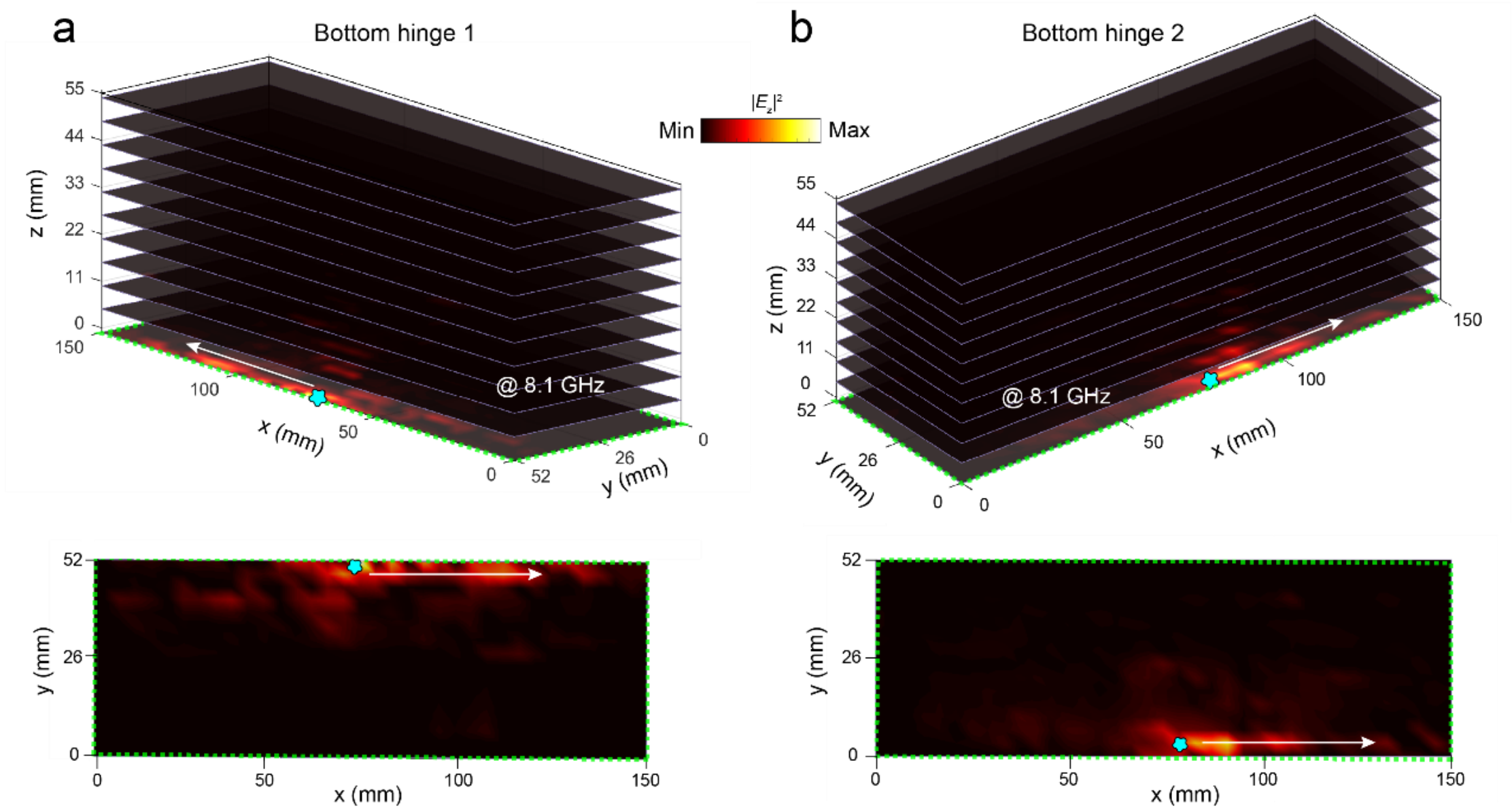

Supplementary Fig. 9 | Measured electric field distributions of the antichiral hinge states at the two bottom hinges. a, b 3D (upper panel) and top (lower panel) views of the measured electric field distributions of the antichiral hinge states at the two bottom hinges.

9. Experimental Setup

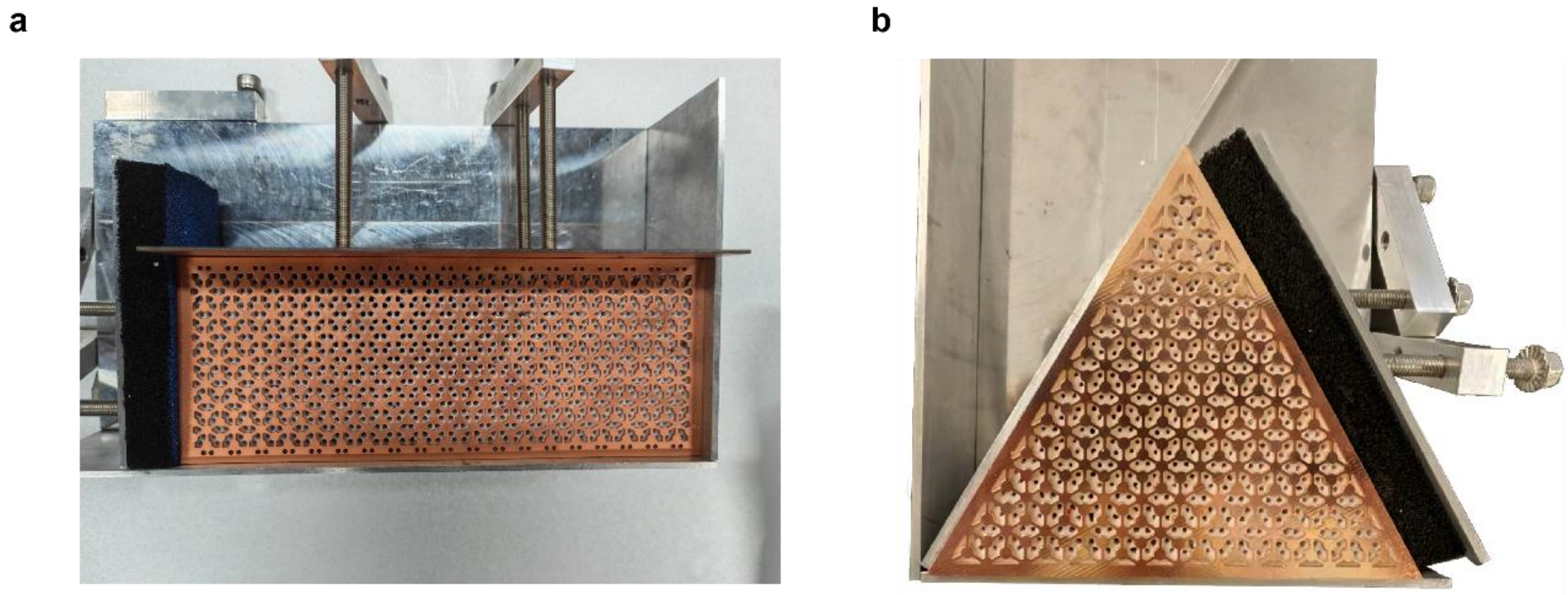

Supplementary Fig. 10 | Photographs of the experimental setups. a Photograph of the experimental setup used for characterizing the antichiral hinge states and evaluating their topological robustness against intentionally introduced lattice defects. **b** Photograph of the experimental setup for the gyromagnetic photonic crystal with sharp corners. Two boundaries of the sample are enclosed by perfect electric conductors (PECs), while absorption boundary conditions are applied to the third.

10. Robustness of the Antichiral Hinge States against Structural Defects

As discussed in the main text (Figs. 5d–f), we further examined the robustness of the measured transmission spectrum and dispersion of the antichiral hinge states by intentionally introducing a local structural defect by removing a gyromagnetic rod and a pair of magnets. As shown in Supplementary Fig. 11a, the measured transmission spectra retain clear nonreciprocal transport characteristics. The corresponding projected band dispersions are resolved in momentum space in Supplementary Fig. 11b. Together, these experimental results align well with those in the absence of local structural defects, providing direct evidence for the robustness of the antichiral hinge states to local structural perturbations.

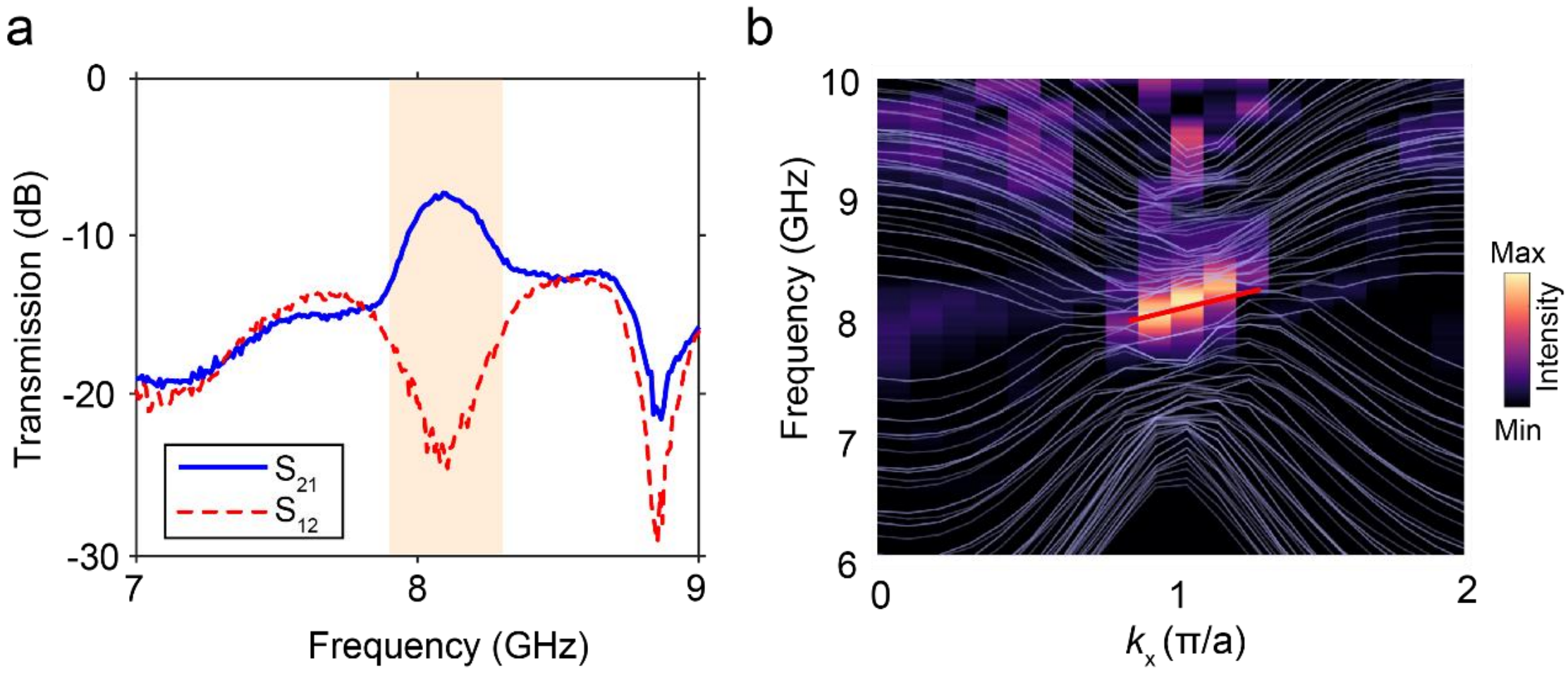

Supplementary Fig. 11 | Measured transmission spectrum and dispersion of the antichiral hinge states with a structural defect. a Measured forward (S_{21}) and backward (S_{12}) transmission spectra of the antichiral hinge states with a structural defect, showing nonreciprocal transport. **b** Measured (colormaps) and simulated (red line) dispersions of the antichiral hinge states with a structural defect.

11. Photonic Device Applications Based on Antichiral Hinge States.

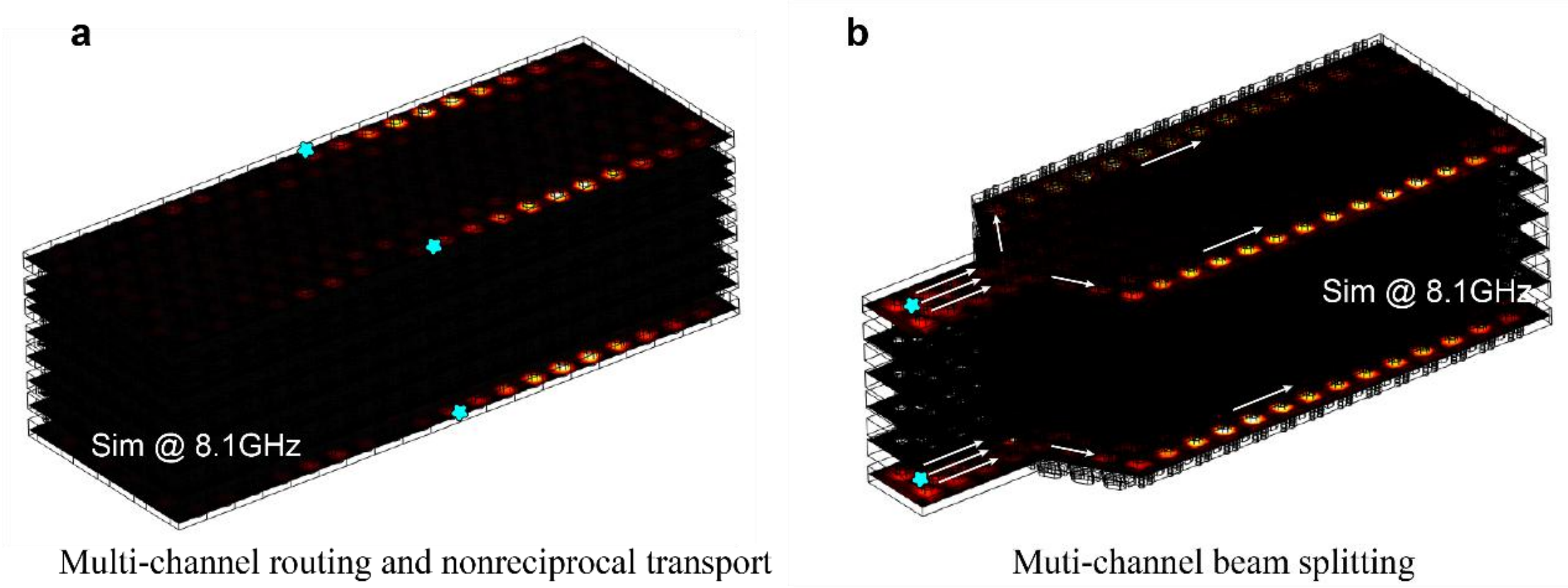

Supplementary Fig. 12 | Photonic device applications based on antichiral hinge states. a Multi-channel routing and nonreciprocal transport along four parallel hinges. **b** Multi-channel beam splitter with co-propagating outputs.
