# Supplementary material for: Observation of Antichiral Hinge States in a Three-dimensional Gyromagnetic Photonic Crystal
Source: Nat Commun. 2026 Aug 26;17:10186. doi: 10.1038/s41467-026-77286-6 (PMC13612583; doi:10.1038/s41467-026-77286-6)
Supplement: Supplementary file 2 [file 41467_2026_77286_MOESM2_ESM.pdf]

nature portfolio

Peer Review File

Observation of Antichiral Hinge States in a Three-dimensional Gyromagnetic Photonic Crystal

Corresponding Author: Professor Zhen Gao

Parts of this Peer Review File have been redacted as indicated to remove third-party material.

This file contains all reviewer reports in order by version, followed by all author rebuttals in order by version.

Version 0:

Reviewer comments:

Reviewer #1

(Remarks to the Author)
The authors implement a three-dimensional modified Haldane model with dimerized interlayer couplings in a gyromagnetic photonic crystal, and theoretically construct and experimentally observe the antichiral hinge states. This work presents timely and conceptual advances by filling an important research gap, given that previous investigations of antichiral states have been confined only to first-order topological phases. The manuscript is well structured with clear logic, and is supported by solid theoretical calculations and consistent experimental results. Overall, this work is well qualified for publication in Nature Communications.
I have a few minor questions for the authors to consider:
1.In addition to the observed antichiral hinge states, antichiral surface states also exist on the crystal(Fig.3c). What unique properties do these antichiral surface states possess compared with conventional topological surface states reported in previous studies?
2.How robust are the present antichiral hinge states against structural defects? For instance, when certain defects are introduced at the sample boundaries, will the transmission spectra(Fig.4b,c), electric field distributions (Fig.4d,e) and band dispersions (Fig.4f,g) of antichiral hinge states be obviously altered?
3.As a new type of higher-order topological state, do the antichiral hinge states hold any unique application prospects?

Reviewer #2

(Remarks to the Author)
The manuscript “Observation of Antichiral Hinge States in a Three-dimensional Gyromagnetic Photonic Crystal” by Wang et. al. reports the experimental observation of antichiral hinge states in a three-dimensional gyromagnetic photonic crystal. The system consists of a 2D modified Haldane model that supports first-order antichiral edge states, with these 2D layers stacked in an SSH-type coupling configuration along the third dimension. The system supports surface states, leading to four antichiral hinge states: two on the top layer and two on the bottom layer. Nonreciprocal propagation in the same direction along the four parallel hinges, as well as the corresponding tilted dispersions, were observed using microwave near-field mapping. The results are sound; however, the broader impact and significance are not clearly addressed.
(1) From a physics perspective, why are the antichiral hinge states reported in this work important, and what new physics do they reveal beyond the existing literature?
(2) Methodologically, the authors state that realizing antichiral hinge states is challenging. What are the main challenges? Compared with gyromagnetic photonic crystals studied in previous works, what technical advances are achieved in the current experimental setup?
(3) The SSH stacking configuration appears relatively straightforward, and one naturally obtains isolated top and bottom layers with edge states. The intermediate layers seem unimportant and could potentially be removed or replaced by any trivial insulating bulk. Therefore, the advantages of antichiral hinge states over 2D antichiral edge states are not clearly demonstrated.
(4) The authors mention possible applications, such as multidimensional transport and 3D photonic devices, but a more detailed analysis would help clarify the impact of this work, especially regarding applications that are unique to 3D antichiral hinge states.

Overall, based on the current material at hand, I cannot make a definite recommendation. I invite the authors to address these questions together with the following minor concerns:
a) Regarding the topological invariant, p_y is quantized within the region bounded by the outer nodal surfaces (NSs). I assume the NSs originate from the coupling along the z-direction, whereas p_y is defined from H_xy. Why does its quantization depend on the position of the NSs?
b) Following question (a), why does the red line in Fig. 2(e) appear not to reach the outer boundary of the NSs?
c) The usage of “Fig. x” and “Figure x” should be made consistent throughout the manuscript.
d) Can the NSs and nodal lines (NLs) be measured directly in such systems?
e) If possible, experimental verification of robustness would be preferable to numerical simulations.

Reviewer #3

(Remarks to the Author)
In this manuscript, Wang and coauthors experimentally demonstrate higher-order 1D antichiral hinge states in a 3D gyromagnetic photonic crystal. Its 3D structure appears to arise from a z-directional SSH-like stack of 2D antichiral semimetals. The emergence of kx hinge states is explained by the combined effects of nontrivial out-of-plane pz polarization and in-plane py polarization. Overall, the current work is novel and timely, and the results are correct and reasonable. I recommend publishing this manuscript in Nature Communications after the authors address the following issues.

1. In the introduction, the authors describe chiral hinge states as “propagating nonreciprocally in opposite directions along two pairs of parallel hinges”, citing Refs [20-22] about photonic axion insulators. However, this transport behavior differs from non-coplanar chiral hinge states characteristic of axion insulators, as the latter do not host parallel hinge transport on the surfaces. The chiral hinge states shown in Fig. 1(c) are more like those realized in the Chern-insulator stack with SSH modulation reported in PRB 111, L041112 (2025), rather than being directly related to axion cases.

2. The theoretical analysis is based on a 3D tight-binding model with quite different parameter values: t1=1, t4=0.11, but t3~0.01. Such a choice leads to an extremely weak interlayer coupling, which can also be seen in Fig. 2c and Fig. 3b with flat bands along the KH, K’H’, and gamma-A directions. Thus, one may doubt the topological properties remain unchanged even when t3=0. Then, the difference between antichiral hinges in 3D and antichiral edges in 2D seems marginal.

3. The measured transmission in Fig. 4(b-c) shows relatively high values over 7-9 GHz except the hinge state window. However, the measured dispersions in Fig. 4(f-g) are unexpectedly clean, with nearly no background states aside from the narrow hinge state window (7.9-8.3 GHz). It is unlike previously semimetal experiments (Refs. [15-17]). The authors would like to clarify this point.

4. The red/blue color lines in Fig. 4 are somewhat confusing and inconsistent. The authors may consider using dashed lines to plot S12 parameters.

5. In Supplemental Material Fig. S1, severe overlap of data hinders readability. And, it would be better if the authors could explain how to technically distinguish the overlapping bulk, surface, and hinge states.

Version 1:

Reviewer comments:

Reviewer #1

(Remarks to the Author)
The authors have provided thorough and satisfactory responses to all my queries. I am therefore happy to recommend this manuscript for publication in Nature Communications.

Reviewer #2

(Remarks to the Author)
Overall, I think the authors have addressed most of my concerns in the revised manuscript. I can recommend its publication in principle. However, I would like to raise one remaining issue regarding my previous Comment 3.

The hinge states discussed in this work are extrinsic, meaning that they can be removed by adding additional surfaces or hinges. Consequently, their topology is determined by the topological properties of the 2D surface states rather than by an intrinsic 3D bulk topological invariant. This is consistent with the well-established concept of surface-obstructed topology. Therefore, I believe the statement in the manuscript that the hinge modes are "protected by the 3D band topology" is somewhat misleading and should be revised. A more accurate description would clarify that the hinge states originate from the topology of the gapped surfaces (or the surface-obstructed topology) rather than from an intrinsic higher-order bulk topological phase.

Reviewer #3

(Remarks to the Author)
The authors have well addressed my concerns arising in the first round of review. I recommend its publication in Nature Communications.

Open Access This Peer Review File is licensed under a Creative Commons Attribution 4.0 International License, which permits use, sharing, adaptation, distribution and reproduction in any medium or format, as long as you give appropriate credit to the original author(s) and the source, provide a link to the Creative Commons license, and indicate if changes were made.
In cases where reviewers are anonymous, credit should be given to 'Anonymous Referee' and the source.
The images or other third party material in this Peer Review File are included in the article's Creative Commons license, unless indicated otherwise in a credit line to the material. If material is not included in the article's Creative Commons license and your intended use is not permitted by statutory regulation or exceeds the permitted use, you will need to obtain permission directly from the copyright holder.
To view a copy of this license, visit https://creativecommons.org/licenses/by/4.0/

Response Letter to Referees

We are grateful for the constructive comments from three referees on our manuscript (NCOMMS-26-026679). In the text below, the referee's comments are quoted in *italics* and are followed by the corresponding detailed response. We also revised the manuscript and the supplementary materials based on the referee's comments and suggestions, and these updates are highlighted in blue in those files. In the text below, references to these updates are also highlighted.

GENERAL COMMENTS FROM REFEREE #1:

The authors implement a three-dimensional modified Haldane model with dimerized interlayer couplings in a gyromagnetic photonic crystal, and theoretically construct and experimentally observe the antichiral hinge states. This work presents timely and conceptual advances by filling an important research gap, given that previous investigations of antichiral states have been confined only to first-order topological phases. The manuscript is well structured with clear logic, and is supported by solid theoretical calculations and consistent experimental results. Overall, this work is well qualified for publication in Nature Communications.

Response from Authors:

We sincerely thank Referee #1 for taking the time to review our manuscript and for providing such a highly positive and encouraging evaluation. In the following, we will address Referee #1's comments point-by-point.

SPECIFIC COMMENTS FROM REFEREE #1:

Referee #1 -- Comment 1:

In addition to the observed antichiral hinge states, antichiral surface states also exist on the crystal (Fig.3c). What unique properties do these antichiral surface states possess compared with conventional topological surface states reported in previous studies?

Response from Authors:

We thank Referee #1 for this insightful question. The most prominent and distinctive property of antichiral surface states, compared to conventional topological surface states (such as the chiral surface states in three-dimensional Chern insulators), is that they co-propagate along parallel surfaces. As shown in Fig. R1a, conventional chiral surface states propagate in opposite directions on two parallel surfaces [Nature 609, 925–930 (2022)]. In stark contrast, antichiral surface states propagate in the same direction on two parallel surfaces (i.e., they co-propagate), as illustrated in Fig. R1b [Nat. Commun. 14, 1991 (2023)].

As the Referee #1 correctly noted, we did not elaborate extensively on these antichiral surface states (Fig. R2) in the original manuscript. This is primarily because the fundamental properties of first-order antichiral surface states have been well established and thoroughly investigated in several excellent prior works. [Nat. Commun. 14, 1991 (2023); Nat. Commun. 14, 2027 (2023)], what we focus on in this manuscript is the

higher-order antichiral hinge states, which have never been experimentally observed yet.

[FIGURE REDACTED]

Fig. R1 | Schematic comparison between conventional chiral and antichiral topological surface states. a Chiral surface states. **b** Antichiral surface states.

To make this point clear, we have added the following discussions in the revised manuscript in lines 130-131 on Page 4:

"The simulated eigenmode profiles of the fourfold-degenerate antichiral hinge states at 8.1 GHz, presented in Fig. 3d, demonstrate that electromagnetic waves are tightly localized at the four parallel hinges (see Supplementary Material, Sec. 7, for the eigenfield profiles of the antichiral surface states)."

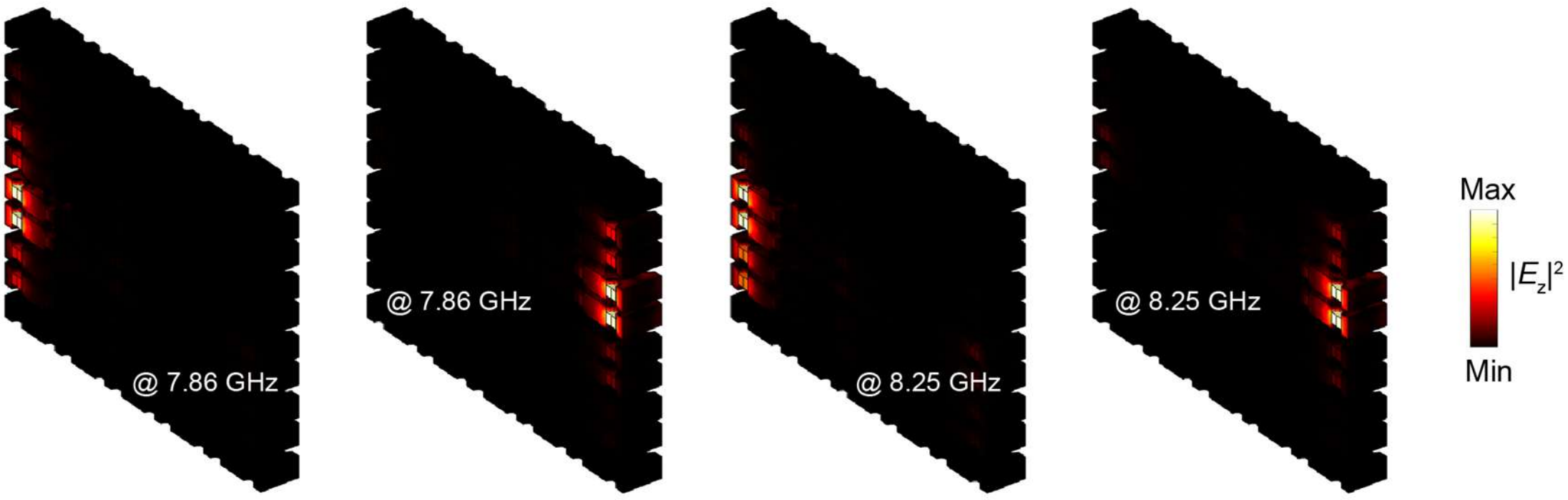

Fig. R2 | Simulated electric field distribution of the eigenmode profiles of the antichiral surface states at $\boldsymbol{k_x = \pi/a}$**.**

Fig. R2 has been adopted as Fig. S6 in the revised Supplementary Materials.

<u>Referee #1 -- Comment 2:</u>

How robust are the present antichiral hinge states against structural defects? For instance, when certain defects are introduced at the sample boundaries, will the transmission spectra (Fig.4b,c), electric field distributions (Fig.4d,e) and band dispersions (Fig.4f,g) of antichiral hinge states be obviously altered?

<u>Response from Authors:</u>

We thank Referee #1 for these constructive comments. Following your valuable suggestion, we have performed a comprehensive new set of experiments to test the

robustness of the antichiral hinge states. Specifically, we intentionally introduced a structural defect by removing a gyromagnetic rod directly along the path of the antichiral hinge states. We then measured the transmission spectra, band dispersions, and near-field electric field distributions of the antichiral hinge states. As shown in Fig. R3, the measured transmission spectra, band dispersions, and electric-field distributions of the antichiral hinge states remain almost unchanged, thereby unambiguously verifying their topological robustness.

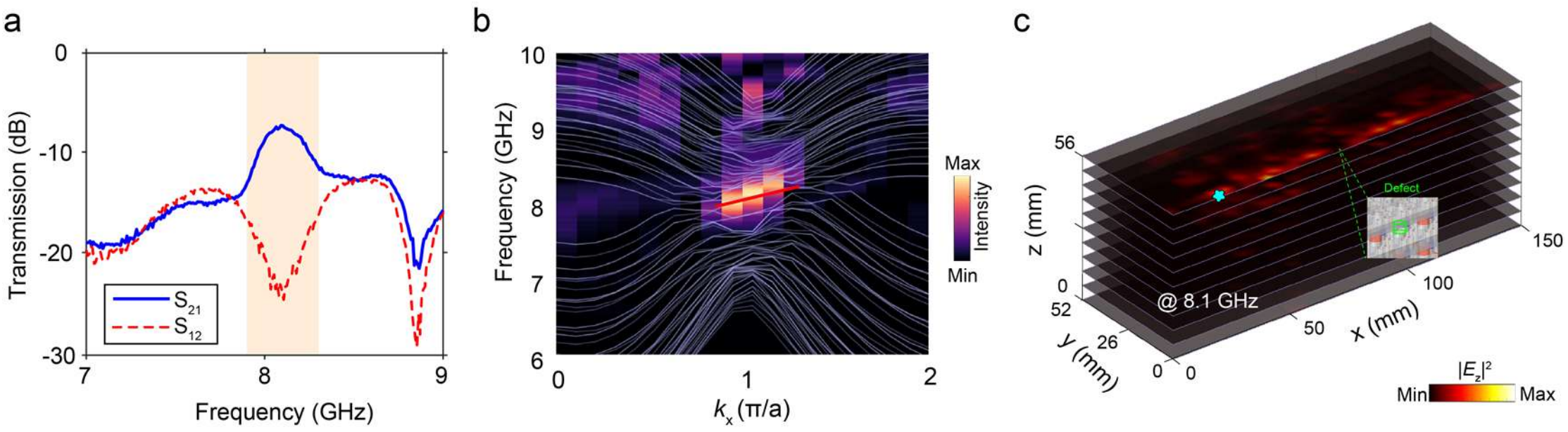

Fig. R3 | Experimental demonstration of the robustness of antichiral hinge states. **a** Measured forward (S_{21}) and backward (S_{12}) transmission spectra of the antichiral hinge states with a structural defect, showing nonreciprocal transport. **b** Measured (colormaps) and simulated (red line) dispersions of the antichiral hinge states with a structural defect. **c** Measured electric field distributions of the antichiral hinge states with a structural defect.

Furthermore, in response to the comments from Reviewer #2, we have also experimentally tested the robustness of antichiral hinge states against sharp corners. To make this point clear, we have added a new section (Experimental demonstration of the robustness of antichiral hinge states) in the revised manuscript:

"Finally, we investigate the robustness of the antichiral hinge states. We first designed and fabricated a 3D gyromagnetic photonic crystal incorporating a sharp corner, as shown in Fig. 5a. Perfect-electric-conductor boundary conditions were imposed on two surfaces of the sample, whereas an absorbing boundary condition was applied to the third (see the experimental setup in Supplementary Material, Sec. 9). The electric-field distributions obtained from full-wave simulations (Fig. 5b) and microwave near-field measurements (Fig. 5c) show that the antichiral hinge states smoothly navigate the sharp corner without discernible backscattering. We further introduced a lattice defect along their propagation path by removing a YIG rod together with its pair of permanent magnets, as shown in Fig. 5d. Both the simulated (Fig. 5e) and measured (Fig. 5f) electric-field profiles demonstrate that the hinge states circumvent the defect with negligible attenuation. The measured transmission spectra and dispersions in the presence of the structural defect are provided in Supplementary Material, Sec. 10. Together, these results confirm the topologically protected robustness of the antichiral hinge states."

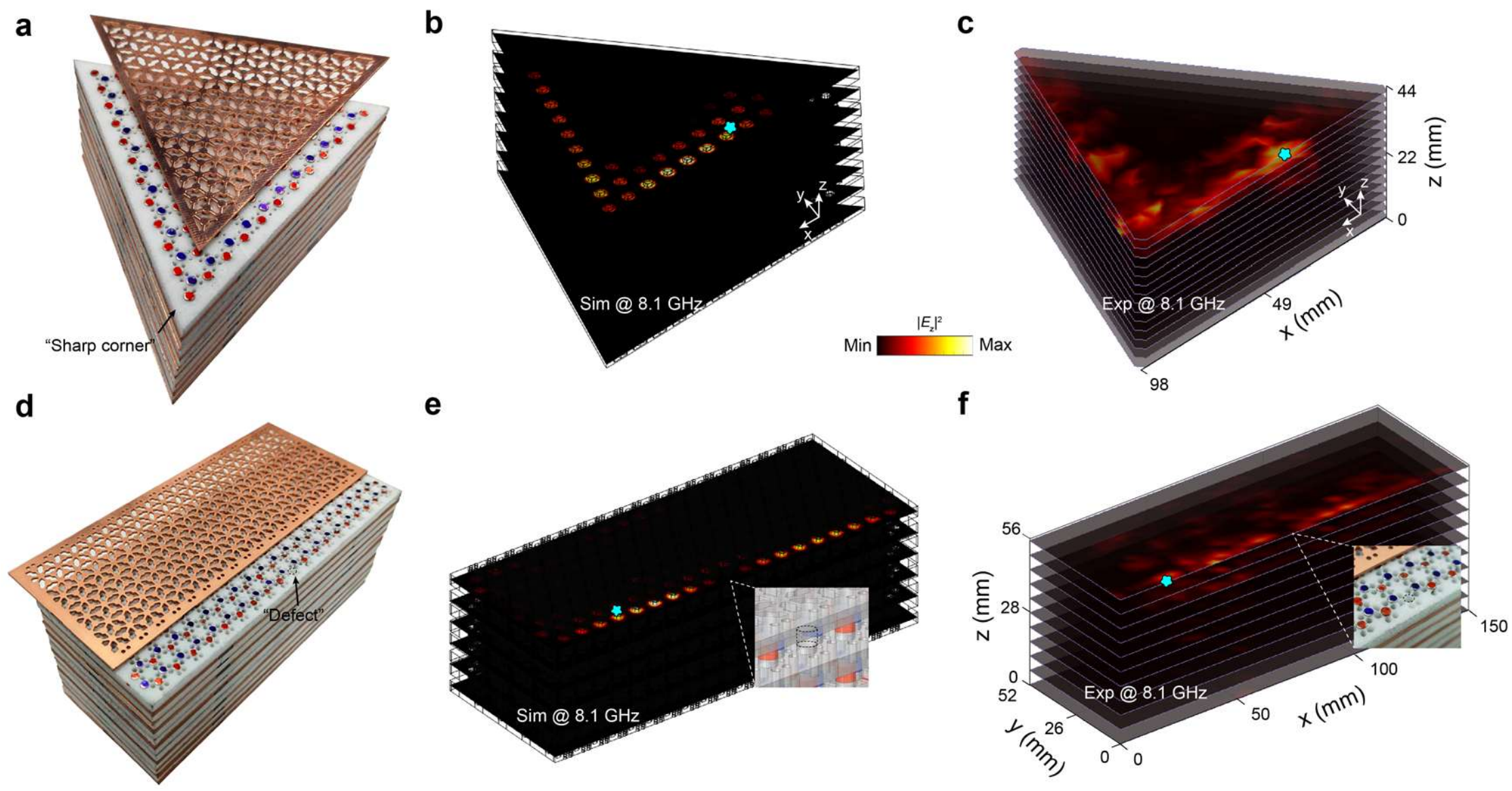

Fig. 5 | Experimental demonstration of the robustness of antichiral hinge states against sharp corners and structural defects. a Photograph of a fabricated 3D gyromagnetic photonic crystal with sharp corners. **b, c** Simulated (b) and measured (c) electric field distributions of the antichiral hinge states route around a sharp corner smoothly without backscattering. **d** Photograph of a fabricated 3D gyromagnetic photonic crystal containing a lattice defect in the top hinge. **e, f** Simulated (e) and measured (f) electric field distributions of the antichiral hinge states propagate through a lattice defect without backscattering.

Fig. R3c has been included in the revised manuscript (now Fig. 5f).

Figs. R3a and R3b have been added to the revised Supplemental Material as Fig. S10 with the following discussion:

"As discussed in the main text (Figs. 5d–f), we further examined the robustness of the measured transmission spectrum and dispersion of the antichiral hinge states by intentionally introducing a local structural defect by removing a gyromagnetic rod and a pair of magnets. As shown in Fig. S10a, the measured transmission spectra retain clear nonreciprocal transport characteristics. The corresponding projected band dispersions are resolved in momentum space in Fig. S10b. Together, these experimental results match well with those in the absence of the local structural defects, providing direct evidence for the robustness of the antichiral hinge states against local structural perturbations."

Referee #1 -- Comment 3:

As a new type of higher-order topological state, do the antichiral hinge states hold any unique application prospects?

Response from Authors:

We sincerely thank Referee #1 for this constructive comment. As a new type of higher-

order topological state, the antichiral hinge states reported in our work feature a unique unidirectional co-propagation mechanism, enabling specific applications in 3D nonreciprocal photonic devices.

1. Robust one-way waveguides: Since the antichiral hinge states exhibit robustness against sharp corners and structural defects, they can be used to realize robust one-way waveguides. As shown in Fig. R4, the antichiral hinge states can smoothly route along complex, curved 3D paths (Fig. R4a) and around structural defects (Fig. R4b) without backscattering.

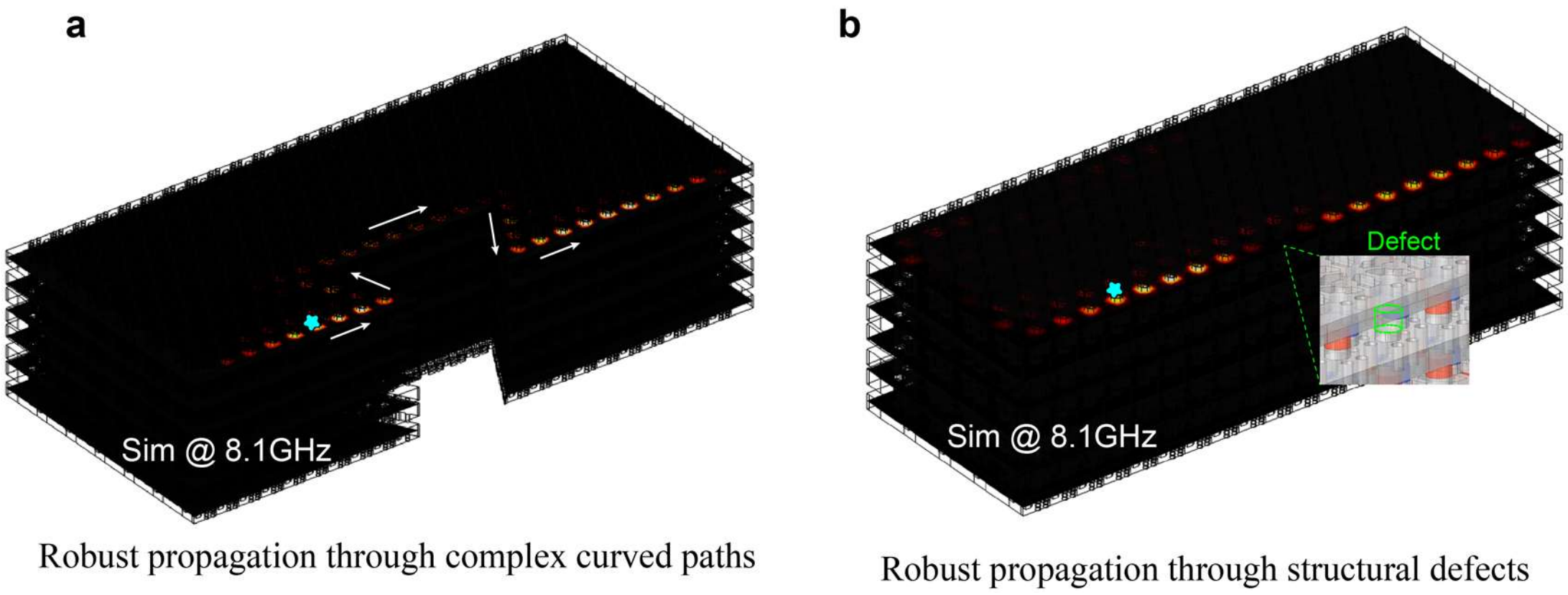

Fig. R4 | Robust one-way waveguide based on antichiral hinge states. a Robust propagation along complex, curved 3D paths. **b** Robust propagation through structural defects.

2. 3D multi-channel one-way waveguide and splitter: Since the antichiral hinge states can propagate in the same direction simultaneously along four parallel hinges, we can use them to design a 3D multi-channel one-way waveguide and splitter, as shown in Fig. R5. This parallel co-propagation capability enhances the data capacity and integration density of 3D photonic devices.

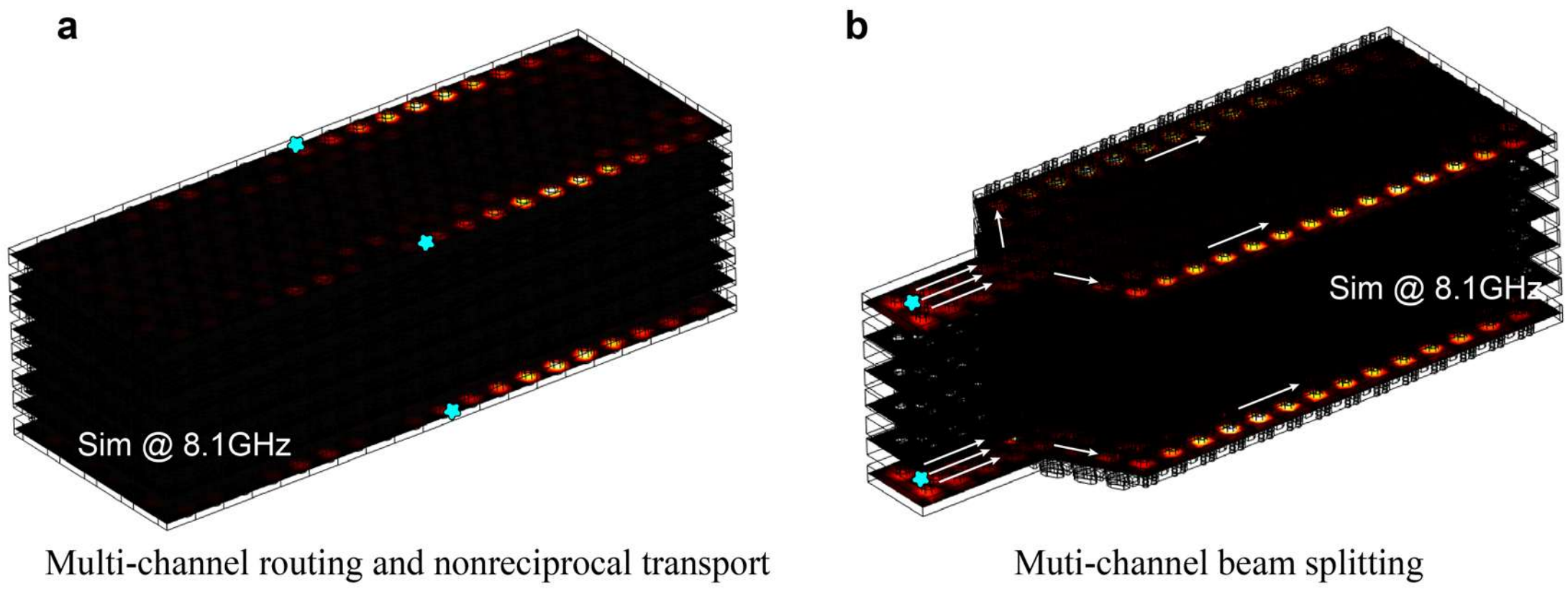

Fig. R5 | Photonic device applications based on antichiral hinge states. a Multi-channel routing and nonreciprocal transport along four parallel hinges. **b** Multi-channel beam splitter with co-propagating outputs.

To make this point clear, we have added the following discussion in the revised manuscript:

“As a 3D form of topologically protected one-way electromagnetic transport, antichiral hinge states offer new opportunities for the development of nonreciprocal photonic devices (see details in the Supplementary Material, Sec. 11).”

The Fig. R5 has been added to the revised Supplementary Materials as Fig. S11.

GENERAL COMMENTS FROM REFEREE #2:

The manuscript "Observation of Antichiral Hinge States in a Three-dimensional Gyromagnetic Photonic Crystal" by Wang et. al. reports the experimental observation of antichiral hinge states in a three-dimensional gyromagnetic photonic crystal. The system consists of a 2D modified Haldane model that supports first-order antichiral edge states, with these 2D layers stacked in an SSH-type coupling configuration along the third dimension. The system supports surface states, leading to four antichiral hinge states: two on the top layer and two on the bottom layer. Nonreciprocal propagation in the same direction along the four parallel hinges, as well as the corresponding tilted dispersions, were observed using microwave near-field mapping. The results are sound; however, the broader impact and significance are not clearly addressed.

Response from Authors:

We thank Referee #2 for the insightful comments and for acknowledging that our theoretical and experimental results are sound. To address the broader impact and significance of our work, we have substantially strengthened the discussion of its broader impact, unique physical significance, and application prospects in the revised manuscript and supplementary materials. Specifically, we have (i) recalculated the band topology using the nested Wilson loop method for the full three-dimensional Hamiltonian; (ii) identified the nodal surfaces via isofrequency calculations based on Green's functions; (iii) performed additional experiments demonstrating the robustness of antichiral hinge states against structural defects and sharp corners; and (iv) expanded the discussion on the distinctive application prospects of the antichiral hinge states, including three-dimensional robust multi-channel wave routing and beam splitting.

In the following, we will address Referee #2's specific comments point-by-point.

SPECIFIC COMMENTS FROM REFEREE #2:

Referee #2 -- Comment 1:

From a physics perspective, why are the antichiral hinge states reported in this work important, and what new physics do they reveal beyond the existing literature?

Response from Authors:

We thank Referee #2 for this insightful question. Our work reveals new aspects beyond the existing literature in the following three aspects:

1. Experimentally extending antichirality to higher-order topological phases for the first time. While antichiral states with their unique co-propagating modes along parallel boundaries have attracted considerable attention, all previous experimental realizations have been limited to first-order topological phases, such as antichiral edge states in 2D systems and antichiral surface states in 3D systems [Phys. Rev. Lett. 125, 263603 (2020); Nat. Commun. 14, 1991 (2023); Nat. Commun. 14, 2027 (2023)]. In this work, we experimentally realized higher-order antichiral hinge states in a 3D gyromagnetic photonic crystal, thereby extending antichirality to higher-order topological phases for the first time.

2. Discovering an exotic bulk-boundary correspondence of higher-order nodal

manifolds. To date, studies of 3D higher-order topological semimetals have been predominantly restricted to zero-dimensional nodal points, such as Weyl and Dirac points [Nat. Commun. 14, 6636 (2023); Phys. Rev. B 105, L060101 (2022)]. Extended higher-order nodal manifolds (e.g., nodal lines and nodal surfaces) have remained largely elusive due to the formidable challenge of translating theoretical models into realistic 3D photonic structures. By engineering an unprecedented configuration in which nodal surfaces (NSs) wrap around nodal lines (NLs), our system unveils an exotic bulk-boundary correspondence of higher-order nodal manifolds: the projected NLs are connected by 2D antichiral surface states, whereas the NSs define the phase boundaries that host the 1D antichiral hinge states.

3. Expanding the 3D Haldane model family. From a fundamental perspective, we propose and experimentally realize a 3D modified Haldane model with dimerized interlayer couplings. The Haldane model and its extensions—from the original 2D Haldane model [Phys. Rev. Lett. 106, 093903 (2011)] and modified Haldane model [Phys. Rev. Lett. 125, 263603 (2020)] to the recent 3D Haldane model [Nature 609, 925–930 (2022)] and modified Haldane model [Nat. Commun. 14, 1991 (2023)]—have provided an important theoretical framework for exploring novel topological phases. Our work extends this framework by proposing the first 3D modified Haldane model with dimerized interlayer coupling, which supports higher-order antichiral hinge states that have not yet been observed.

Referee #2 -- Comment 2:

Methodologically, the authors state that realizing antichiral hinge states is challenging. What are the main challenges? Compared with gyromagnetic photonic crystals studied in previous works, what technical advances are achieved in the current experimental setup?

Response from Authors:

We thank Referee #2 for this insightful comment on our methodology. Realizing these antichiral hinge states presents two main challenges: theoretical mapping and experimental implementation.

First, tight-binding models are inherently based on scalar wavefunctions, whereas electromagnetic waves in 3D photonic systems are intrinsically fully vectorial. Due to this fundamental mismatch, the full vectorial nature of 3D Maxwell's equations inevitably leads to complex band dispersions and intricate cross-couplings between neighboring lattice sites [Nat. Mater. 22, 1203 – 1209 (2023); Nat. Commun. 16, 3122 (2025)]. While 2D photonic crystals permit electromagnetic waves to be decoupled into independent TE and TM modes via mirror symmetry—thereby enabling a simplified mapping to scalar wave equations—this decoupling strictly breaks down in 3D systems. Consequently, faithfully reproducing an idealized scalar tight-binding Hamiltonian in a realistic 3D photonic crystal is challenging. In our work, we addressed this challenge through meticulous full-wave design and optimization, ensuring that the photonic crystal's vectorial bands closely mimic the tight-binding results.

Second, in most previous studies on gyromagnetic photonic crystals (such as conventional 2D/3D Haldane models or photonic Chern insulators), time-reversal symmetry is broken by applying a uniform external magnetic field across the entire photonic structure [Nature 461, 772 (2009); Nature 609, 925 (2022)]. This is relatively straightforward to achieve using large electromagnets or Helmholtz coils. However, for the antichiral hinge states in our 3D modified Haldane model with dimerized interlayer coupling, a uniform external magnetic field is insufficient. The model strictly demands site-dependent magnetization on a 3D honeycomb lattice: opposite magnetic fluxes must be applied to the A and B sublattice sites. Generating such a staggered, spatially non-uniform magnetic bias throughout a complex 3D bulk architecture is a formidable challenge. We overcame this challenge by developing a bottom-up magnetization scheme, which constitutes the main technical advance of the present setup. Instead of using a global external magnetic field, each individual yttrium iron garnet (YIG) rod in our structure is sandwiched between a pair of permanent magnets. This site-by-site assembly allows us to precisely and independently control the local magnetic bias at every site across the entire 3D structure. The magnet surfaces are electroplated with a nickel layer, enabling them to act as perfect electric conductors (PECs) in the numerical simulations. This crucial treatment ensures that the magnets do not introduce any extra eigenmodes into the system. Moreover, assembling such a dense 3D array of magnets induces strong mutual magnetic attraction, posing an additional structural challenge. To resolve this, we secured the magnets with a supporting foam having a dielectric constant close to unity (≈1), ensuring the structure remains mechanically stable.

Referee #2 -- Comment 3:

The SSH stacking configuration appears relatively straightforward, and one naturally obtains isolated top and bottom layers with edge states. The intermediate layers seem unimportant and could potentially be removed or replaced by any trivial insulating bulk. Therefore, the advantages of antichiral hinge states over 2D antichiral edge states are not clearly demonstrated.

Response from Authors:

We thank Referee #2 for this comment. We respectfully disagree with the conjecture that the intermediate layers are unimportant and could be removed or replaced by a trivial insulating bulk. To clarify the indispensable role of the intermediate layers and outline the unique advantages of our 3D system, we detail our response from the following three perspectives:

1. Topological invariants of the 3D Hamiltonian: In the original manuscript, we calculated the topological invariants for the in-plane H_{xy} and out-of-plane H_z components separately (Fig. R7a), which inadvertently obscured the 3D nature of the Hamiltonian. To correct this, we have recalculated the topological invariant from the complete 3D Hamiltonian using the nested Wilson loop method.

To ensure the validity of this method, one must first verify the existence of a Wannier gap. We constructed the Wilson loop operator along the k_z direction to obtain the Wannier centers $v_z(k_x, k_y)$. As shown in Fig. R6a, the Wannier bands strictly avoid

crossing across the entire Brillouin zone. Specifically, the Wannier centers are tightly localized near $v_z = 0$ in momentum regions away from the projected nodal surfaces (NSs), whereas they shift toward ± 0.5 in the vicinity of the NSs. Cross-sectional profiles of the Wannier bands at selected transverse momenta (Fig. R6b-f) further confirm the strict absence of band crossings. This gap cleanly splits the Wannier bands into separate sectors.

Based on this properly isolated Wannier basis, we then calculated the nested Wannier-sector polarization $p_y^{v_z}$. As illustrated in Fig. R7b, the nested polarization $p_y^{v_z}$ takes the quantized values 1/2 within the region $k_{s1}<k_x<k_{s2}$, where k_{s1} and k_{s2} denote the inner boundaries of the projected nodal surfaces (NSs). Noted that the cyan-shaded area denotes the region within the NSs where the nested polarization is not well-quantized, similar to the representation in [Phys. Rev. Lett. 132, 186601 (2024)]. This non-trivial quantization unambiguously demonstrates that the antichiral hinge states are globally protected by the 3D bulk topology, completely distinguishing them from the antichiral edge states in 2D systems.

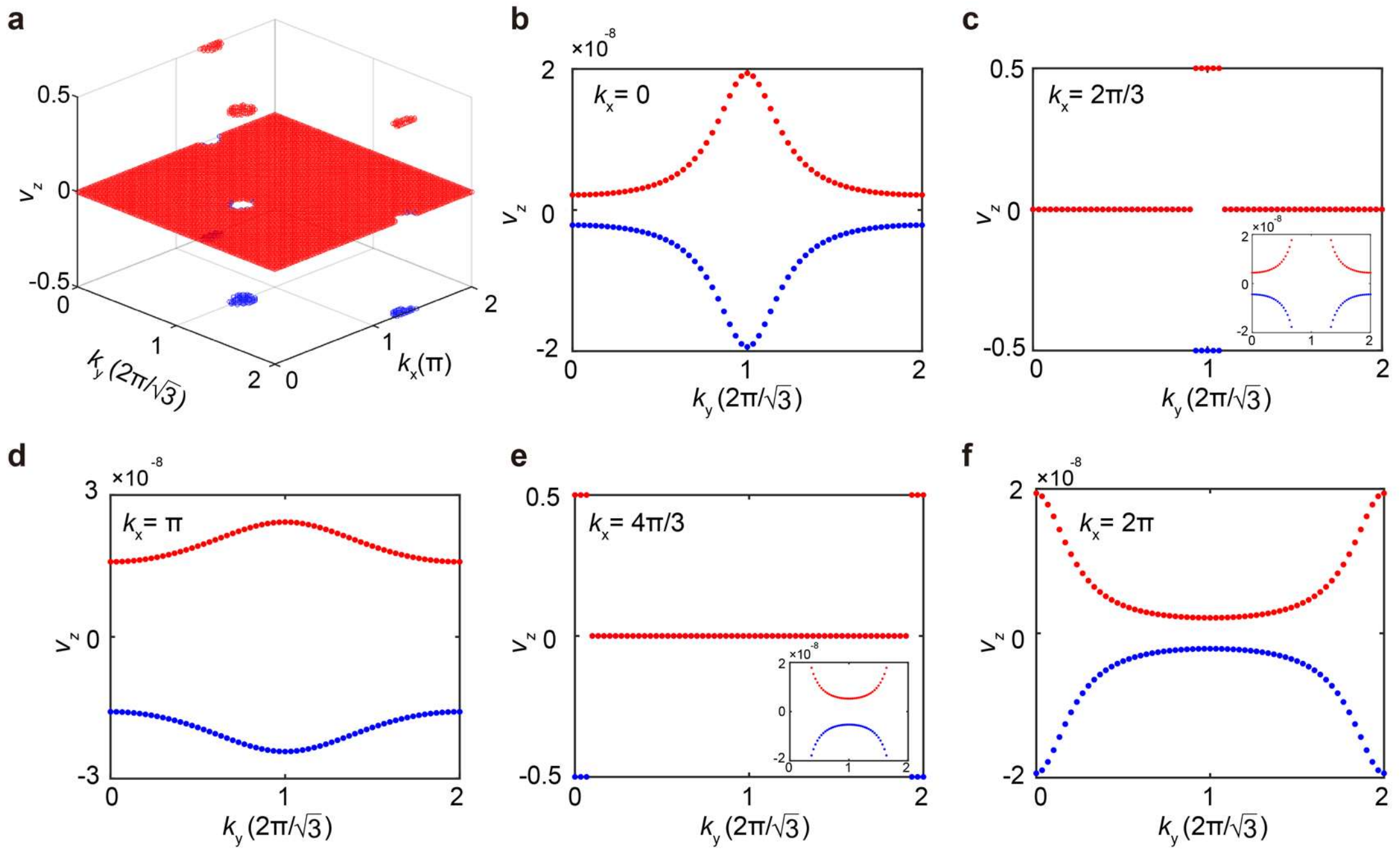

Fig. R6 | Evolution of Wannier centers and the global Wannier band gap. a Three-dimensional distribution of the Wannier centers v_z over the two-dimensional momentum space (k_x, k_y) of the first Brillouin zone. The red and blue markers distinguish the two constituent Wannier bands. **b-f** Cross-sectional profiles of the Wannier bands v_z along the k_y direction at selected transverse momenta: (b) $k_x = 0$, (c) $k_x = \frac{2\pi}{3}$, (d) $k_x = \pi$, (e) $k_x = \frac{4\pi}{3}$, (f) $k_x = 2\pi$. The insets in (c) and (e) provide magnified views of the transition regions, confirming the absence of band crossings. The two Wannier bands remain separated by a finite gap across the entire Brillouin zone, ensuring that the projection onto a single Wannier sector for the subsequent nested Wilson loop calculation is topologically well-defined.

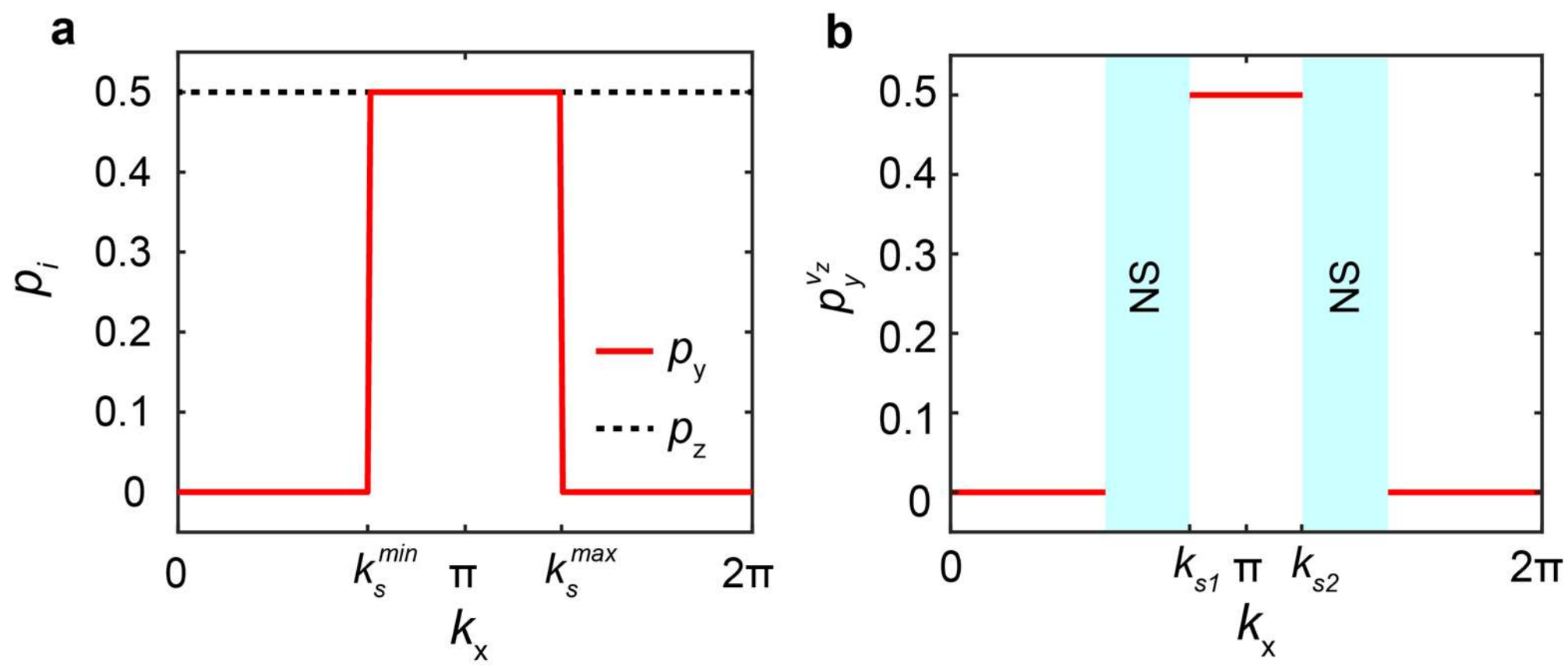

Fig. R7 | Comparison of topological invariants. **a** Previous calculation approach based on separated in-plane and out-of-plane Hamiltonian components. **b** The nested polarization $p_y^{v_z}$ calculated from the full 3D Hamiltonian.

<u>2. The intermediate layers are the origin of the 3D nodal surface and nodal line semimetal phase:</u>

The intermediate layers introduce the k_z-dependent interlayer couplings that are essential for the 3D band topology. These interlayer couplings generate nodal surfaces (NSs) and nodal lines (NLs) in the 3D Brillouin zone, which together form a 3D nodal surface and nodal line semimetal with an "NS-wrapping-NL" configuration. It is the 3D semimetal phase that dictates a hierarchical boundary response: the antichiral surface states bridge the projected NLs, while the antichiral hinge states are bounded by the projected NSs. Such an exotic 3D bulk topology—and the resulting first-order and higher-order antichiral topological phases—cannot exist in a trivial stack of isolated 2D layers.

<u>3. Multi-channel 3D routing capability:</u> The primary advantage of antichiral hinge states in 3D systems over the antichiral edge states in 2D systems is their multi-channel 3D routing capability. A 2D system provides only two copropagating channels confined to a single plane. In contrast, our 3D system supports antichiral hinge (surface) states that propagate along four parallel hinges (two parallel surfaces). This provides a versatile platform for high-capacity, multidimensional wave routing, which is essential for future 3D photonic integrated circuits but unattainable in 2D systems.

To make this point clear, we have added the following discussion in lines 102-111 on Page 4 of the revised manuscript:

"To establish the topological origin of the antichiral hinge states, we use the nested Wilson-loop method to calculate the nested polarization $p_y^{v_z}$ throughout the 3D momentum space (see Supplementary Material, Sec. 5 for the detailed calculation). As shown in Fig. 2f, $p_y^{v_z}$ is quantized to $1/2$ within the momentum interval bounded by the inner projected boundaries of the NSs. This half-quantized polarization is a hallmark of higher-order topology and underlies the bulk–hinge correspondence in our system. Notably, the nontrivial momentum region identified by this quantized 3D invariant coincides precisely with the momentum interval over which the antichiral hinge states appear. This agreement demonstrates that the localized, co-propagating antichiral hinge modes are protected by the 3D higher-order band topology (see Supplementary Material,

Sec. 6 for the theoretical calculation with larger out-of-plane couplings)."

We have updated Fig. 2 in the revised manuscript.

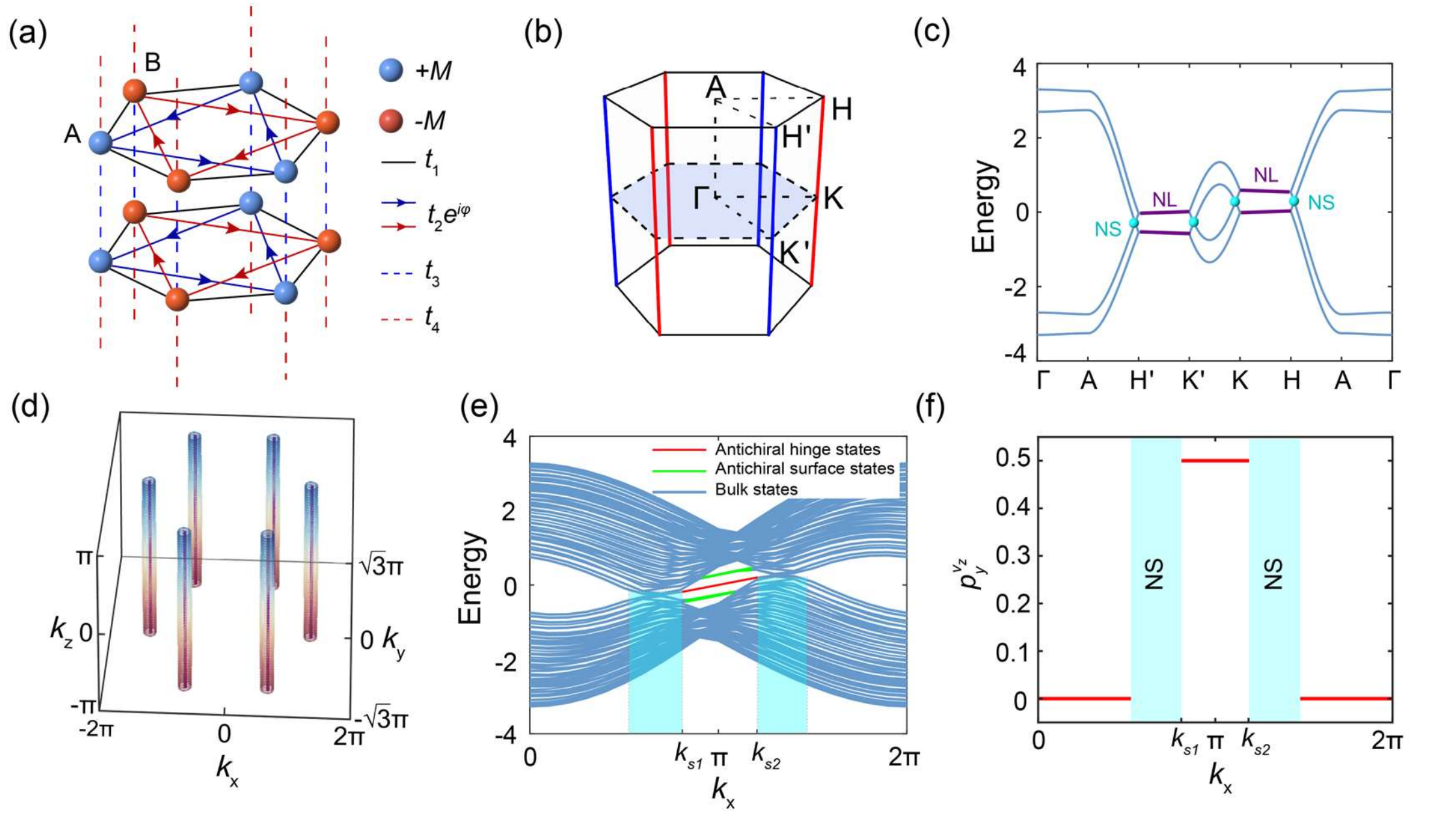

Fig. 2 | Theoretical model. a Schematic of a 3D modified Haldane model with dimerized interlayer couplings. Sublattices A and B are denoted by blue and red spheres, respectively. **b** The 3D BZ. **c** Calculated bulk band structure of the 3D tight-binding model with parameters $M = 0$, $t_1 = 1$, $t_2 = 1/18$, $\varphi = \pi/2$, $t_3 = 0.2t_2$, and $t_4 = 5t_2$. The cyan spheres represent the nodal surfaces, and the purple lines denote the nodal lines. **d** Visualization of the nodal surfaces (colored surfaces) and nodal lines (purple lines) in the 3D momentum space. **e** Projected band dispersions of a 3D modified Haldane model with dimerized interlayer coupling that is finite in the y- and z-directions and periodic in the x-direction. Red and green lines represent the antichiral hinge and surface states, respectively. The cyan-shaded region represents the nodal surfaces. **f** The nested polarization $p_y^{v_z}$ is evaluated along k_x computed via nested Wilson loops for occupied bands .

In the revised Supplementary Materials, we present a detailed description of the nested Wilson loop calculations:

"To characterize the higher-order bulk band topology of the antichiral hinge states, we implement the nested Wilson loop method to calculate the nested polarization $p_y^{v_z}$ based on the 3D tight-binding Hamiltonian.

For a half-filled system with N_{occ} occupied bands (here N_{occ}=2), we first construct the Wilson loop operator $\mathcal{W}_z(k_x, k_y)$ along the k_z direction by taking the path-ordered product of the overlap matrices over a period, defined as $[F_{z,k}]_{mn} = \langle u_{m,k} | u_{n,k+\Delta k_z} \rangle$, where $|u_{m,k}\rangle$ represents the occupied Bloch wavefunctions.

Diagonalizing this out-of-plane Wilson loop operator yields the k_z-directed Wannier centers $v_z^j(k_x, k_y)$ and the corresponding Wannier eigenstates

$|\omega_{z,j}(k_x,k_y)\rangle$ via the $\mathcal{W}_z|\omega_{z,j}\rangle = e^{i2\pi v_z^j}|\omega_{z,j}\rangle$. As illustrated in Fig. S4a, the Wannier bands strictly avoid crossing across the entire Brillouin zone due to the dimerized interlayer couplings along the z-axis. Specifically, the Wannier centers are tightly localized near $v_z = 0$ in momentum regions away from the projected nodal surfaces (NSs), whereas they shift toward ± 0.5 in the vicinity of the NSs. Cross-sectional profiles of the Wannier bands at selected transverse momenta (Fig. S4b-f) further confirm the strict absence of band crossings. This gap cleanly splits the Wannier bands into separate sectors. We then isolate the upper Wannier sector by extracting the specific eigenvector matrix $V_w(k_x,k_y)$ corresponding to the upper branch to serve as our localized Wannier basis.

Subsequently, the Bloch state overlap matrices along the k_y direction, $[F_{y,k}]_{mn}=\left\langle u_{m,k}\middle|u_{n,k+\Delta k_y}\right\rangle$, are calculated across the momentum space. To maintain strict numerical unitarity on the discretized grid and ensure gauge smoothness during propagation, a singular value decomposition is performed on $F_{y,k}$ before projecting it onto the Wannier basis, which systematically constructs the nested overlap matrix $\tilde{F}_{y,k} = V_w{}^{\dagger}F_{y,k}V_w(k+\Delta k_y)$. Finally, the nested Wilson loop operator $\widetilde{\mathcal{W}}_y(k_x)$ is obtained by the product of these projected matrices along the closed k_y loop, $\widetilde{\mathcal{W}}_y(k_x) = \prod_{k_y}\tilde{F}_{y,k}$. Because the occupied subspace has been successfully projected into a single Wannier sector, $\widetilde{\mathcal{W}}_y(k_x)$ reduces to a scalar complex number whose phase directly yields the quantized nested polarization $p_y^{v_z} = \frac{1}{2\pi}\mathrm{Im}[\ln\widetilde{\mathcal{W}}_y(k_x)]$ mod 1."

The Fig. R6 has been added to the revised Supplementary Materials as Fig. S4.

<u>Referee #2 -- Comment 4:</u>

The authors mention possible applications, such as multidimensional transport and 3D photonic devices, but a more detailed analysis would help clarify the impact of this work, especially regarding applications that are unique to 3D antichiral hinge states.

<u>Response from Authors:</u>

We sincerely thank Referee #2 for this constructive comment. As a new type of higher-order topological states, the antichiral hinge states reported in our work feature a unique unidirectional co-propagation mechanism, enabling specific applications in 3D nonreciprocal photonic devices.

<u>1. Robust one-way waveguides</u>: Since the antichiral hinge states exhibit robustness against sharp corners and structural defects, they can be used to realize robust one-way waveguides. As shown in Fig. R4, the antichiral hinge states can smoothly route along complex, curved 3D paths (Fig. R4a) and around structural defects (Fig. R4b) without backscattering.

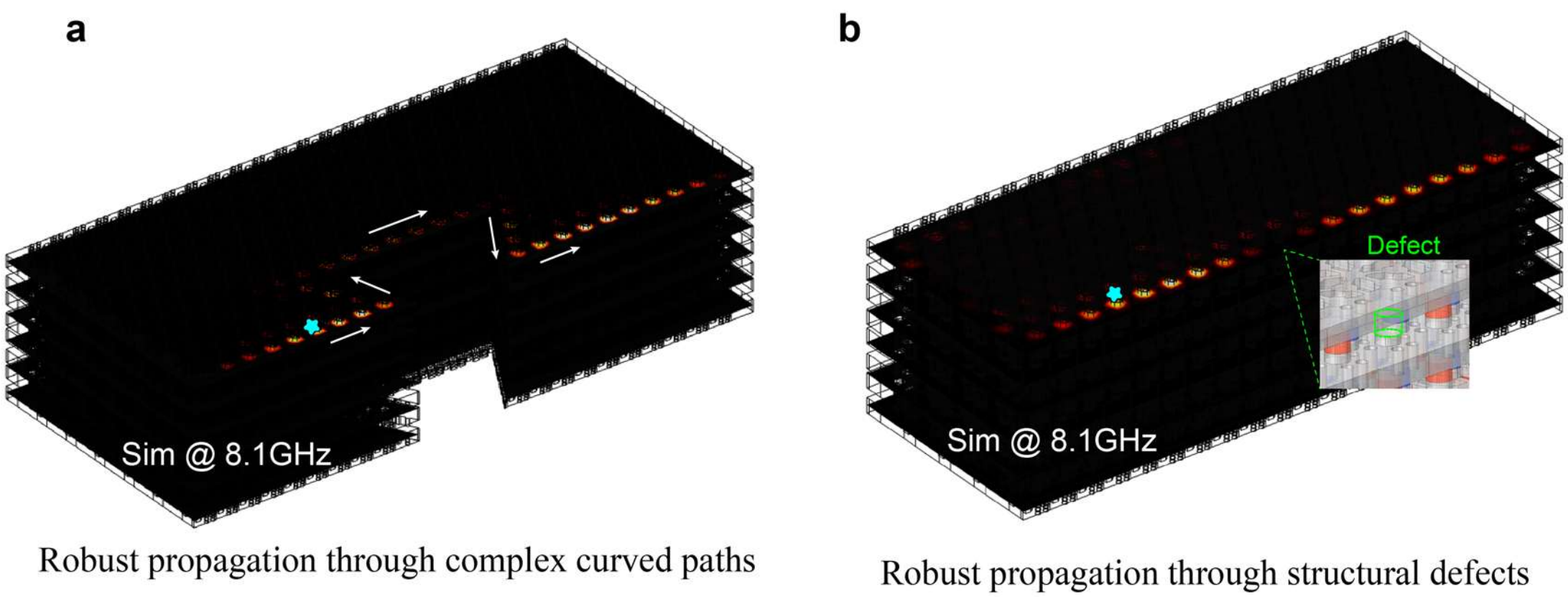

Fig. R4 | Robust one-way waveguide based on antichiral hinge states. a Robust propagation along complex, curved 3D paths. **b** Robust propagation through structural defects.

2. 3D multi-channel one-way waveguide and splitter: Since the antichiral hinge states can propagate in the same direction simultaneously along four parallel hinges, we can use them to design a 3D multi-channel one-way waveguide and splitter, as shown in Fig. R5. This parallel co-propagation capability enhances the data capacity and integration density of 3D photonic devices.

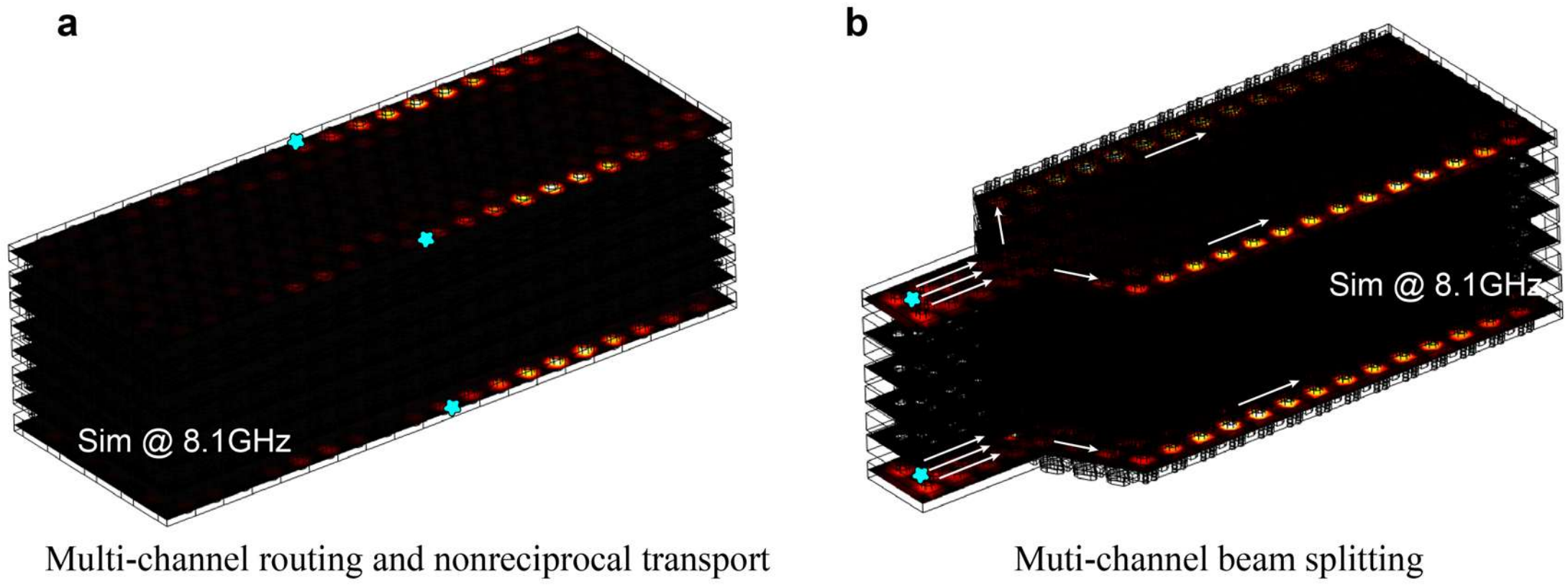

Fig. R5 | Photonic device applications based on antichiral hinge states. a Multi-channel routing and nonreciprocal transport along four parallel hinges. **b** Multi-channel beam splitter with co-propagating outputs.

To make this point clear, we have added the following discussion in the revised manuscript:

"As a 3D form of topologically protected one-way electromagnetic transport, antichiral hinge states offer new opportunities for the development of nonreciprocal photonic devices (see details in the Supplementary Material, Sec. 11)."

The Fig. R5 has been added to the revised Supplementary Materials as Fig. S11.

Referee #2 -- Comment a:

Regarding the topological invariant, p_y *is quantized within the region bounded by the outer nodal surfaces (NSs). I assume the NSs originate from the coupling along the z-*

direction, whereas p_y *is defined from* H_{xy}*. Why does its quantization depend on the position of the NSs?*

Response from Authors:

We sincerely thank Referee #2 for this thought-provoking comment, which highlights an important aspect of our theoretical description.

Quantization relies on the positions of the nodal surfaces (NSs), as they physically represent the topological phase transition points of the entire 3D system. In topological band theory, a quantized invariant can only change its value when the bulk energy gap closes. In our theoretical model, the 3D bulk gap closes at the NSs. Therefore, the projected position of the NSs inherently serves as the exact momentum-space boundary that triggers a change in the topological invariant.

In the previous version of our manuscript, we computed the topological invariants separately for the in-plane H_{xy} and out-of-plane H_z components. In this case, the p_y calculated solely from the 2D Hamiltonian merely labeled the boundaries K and K' ($k_s^{\min}$ and $k_s^{\max}$), which, in fact, obscured the system's genuine 3D nature and created the illusion that p_y was independent of the z-direction coupling.

In response to your comment, we have recalculated the topological invariant using the complete 3D Hamiltonian via the nested Wilson loop method.

As detailed in our response to your Comment 3, applying the nested Wilson loop method to the full 3D Hamiltonian yields a strictly quantized nested polarization $p_y^{v_z}$ of 1/2 exactly within the momentum region $k_{s1}<k_x<k_{s2}$, defined by the projected NSs. This non-trivial quantization proves that the antichiral hinge states are globally protected by the 3D higher-order band topology.

Referee #2 -- Comment b:

Following question (a), why does the red line in Fig. 2(e) appear not to reach the outer boundary of the NSs?

Response from Authors:

We sincerely thank Referee #2 for the careful reading. Indeed, the fourfold-degenerate antichiral hinge states (red line) lie strictly along the inner boundaries rather than the outer boundaries of the NSs.

The strict termination of the antichiral hinge states at the inner boundaries is fundamentally dictated by the definition of the topological invariant. The quantized nested polarization ($p_y^{v_z}$ = 1/2) exists only within the momentum interval between the two inner boundaries of NSs, perfectly corroborating the presence of the antichiral hinge states. Within the projected NSs, without a well-defined quantized invariant to provide topological protection, the system can no longer sustain the hinge modes, thereby truncating them at the inner boundaries.

We have revised the corresponding text in the revised manuscript as follows:

"As shown in Fig. 2f, $p_y^{v_z}$ is quantized to $1/2$ within the momentum interval bounded by the inner projected boundaries of the NSs.

Referee #2 -- Comment c:

The usage of "Fig. x" and "Figure x" should be made consistent throughout the manuscript.

Response from Authors:

We thank Referee #2 for pointing out this formatting inconsistency. We have carefully checked the text and standardized the terminology. All instances of "Figure" have now been uniformly changed to "Fig." throughout the revised manuscript:

"The projected band dispersion of a 3D modified Haldane model with dimerized interlayer couplings—finite in the *y*- and *z*-directions and periodic in the *x*-direction—is presented in Fig. 2e, from which we can see that the tilted four-fold-degenerate hinge dispersions (red line) connect the projections of the frequency-shifted NSs (cyan regions), whereas the tilted surface dispersions (green lines) emanate from the frequency-shifted NLs."

"The simulated bulk band structure of the 3D gyromagnetic photonic crystal (Fig. 3b) closely matches the theoretical results presented in Fig. 2c, further verifying the coexistence of NLs and NSs."

"The simulated eigenmode profiles of the fourfold-degenerate antichiral hinge states at 8.1 GHz, presented in Fig. 3d, demonstrate that electromagnetic waves are tightly localized at the four parallel hinges."

Referee #2 -- Comment d:

Can the NSs and nodal lines (NLs) be measured directly in such systems?

Response from Authors:

We sincerely thank Referee #2 for this insightful question. In principle, the nodal surfaces (NSs) and nodal lines (NLs) can be directly measured by mapping the internal electric field distributions within the 3D bulk using microwave near-field measurements. A 3D spatial FFT of these mapped electric field distributions can directly reconstruct the momentum-space isofrequency contours. Indeed, our experimental setup is physically compatible with such a measurement, as we have intentionally reserved air holes for inserting the probe antenna.

However, the fundamental obstacle to directly resolving the continuous NSs and NLs in our current experimental sample is the strictly limited momentum-space resolution inherent in the spatial Fourier transform of finite-sized samples. Generally, achieving a sufficient resolution in momentum space to clearly distinguish these complex 3D nodal structures requires the real-space lattice to span at least 15 periods along each of the three dimensions. Constructing such a massive 3D gyromagnetic photonic crystal (e.g., 15×15×15 unit cells) would require assembling nearly 28,000 YIG rods and 56,000 biasing permanent magnets, which is unfortunately both experimentally and financially

prohibitive.

To provide rigorous theoretical verification, we calculated the local density of states (LDOS) for a finite-thickness slab using the Green's function method. This yields the momentum-space spectral function, which corresponds to the isofrequency contours obtained from the spatial Fourier transform of the measured electric field distributions.

Fig. R8 presents the theoretically calculated momentum-space LDOS at the nodal energy E = -0.24. At this specific energy, only the nodal structures around the *K-H* path (as depicted in Fig. 2c) are excited. As shown in the k_x-k_y projections (Fig. R8a and Fig. R8d), the white dashed hexagon delineates the boundary of the first Brillouin zone, around whose three vertices the nodal structure clearly emerges. Specifically, the nodal line projects onto the k_x-k_y plane as distinct singular points at these vertices, while the nodal surface manifests as bright continuous rings surrounding them. The geometric profile perfectly corroborates the 3D nodal structure illustrated in Fig. 2d.

In the k_x-k_z cross-sections (Fig. R8b and R8e), we indicate the positions of the nodal surfaces using dashed white lines. It is evident that the bulk spectra exhibit continuous vertical bright walls extending along the entire k_z direction around $k_x = 2\pi/3$. Furthermore, the antichiral surface states are prominently bright at this energy in Fig. R8e, which is in excellent agreement with the projected band dispersion depicted in Fig. 2e.

Finally, the k_y-k_z projections illustrated in Fig. R8c and R8f further confirm the phenomenon that the nodal structure extends continuously along the whole k_z direction around k_y = 0, which is thoroughly consistent with Fig. 2d.

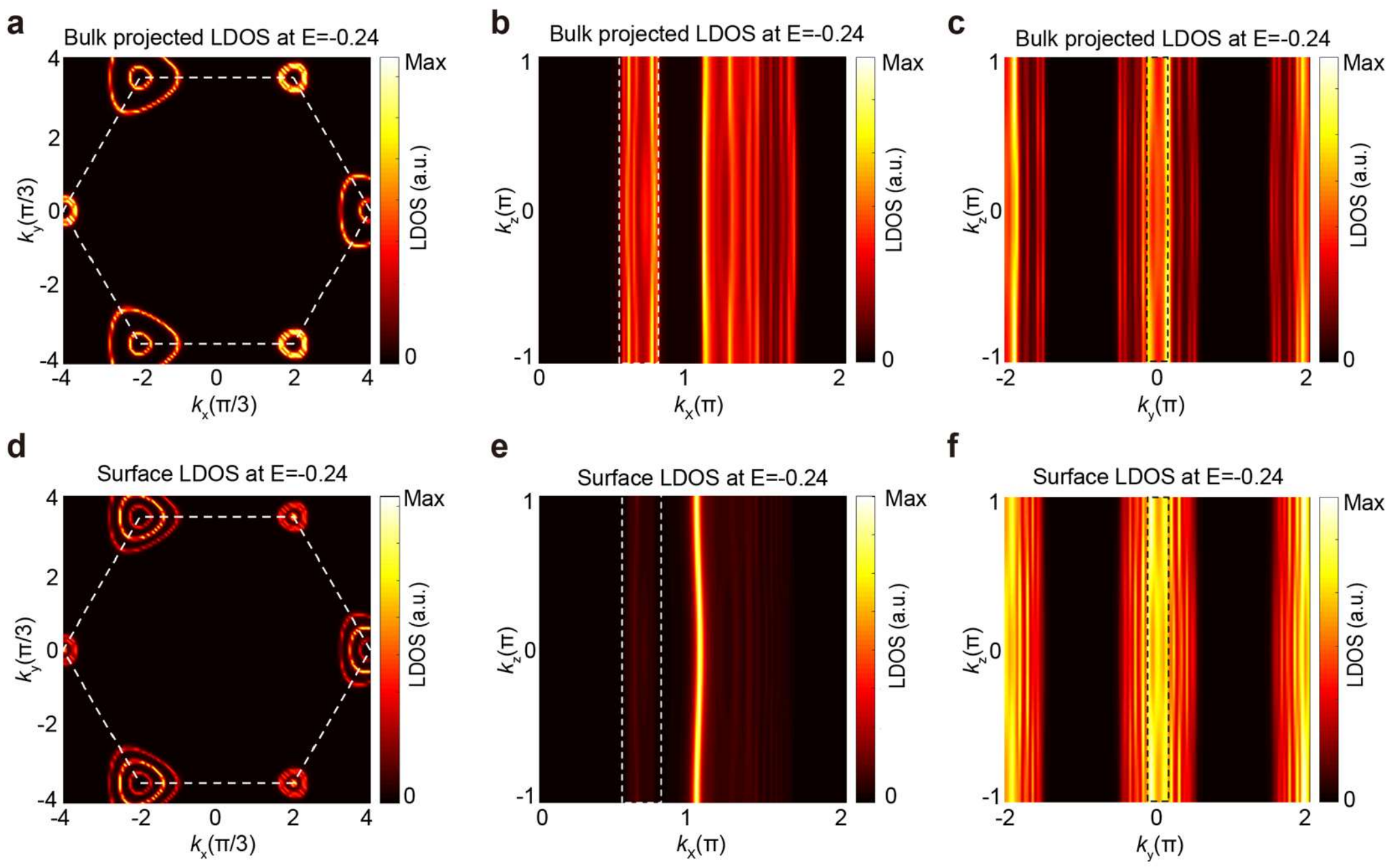

Fig. R8 | Theoretically calculated momentum-space LDOS at the nodal frequency

E = -0.24. a-c Bulk isofrequency contours projected onto the k_x-k_y, k_x-k_z, and k_y-k_z planes, respectively. **d-f** Surface isofrequency contours on the k_x-k_y, k_x-k_z, and k_y-k_z planes, respectively.

Similarly, the corresponding isofrequency contour at E = 0.24 is presented in Fig. R9, exhibiting similar topological features.

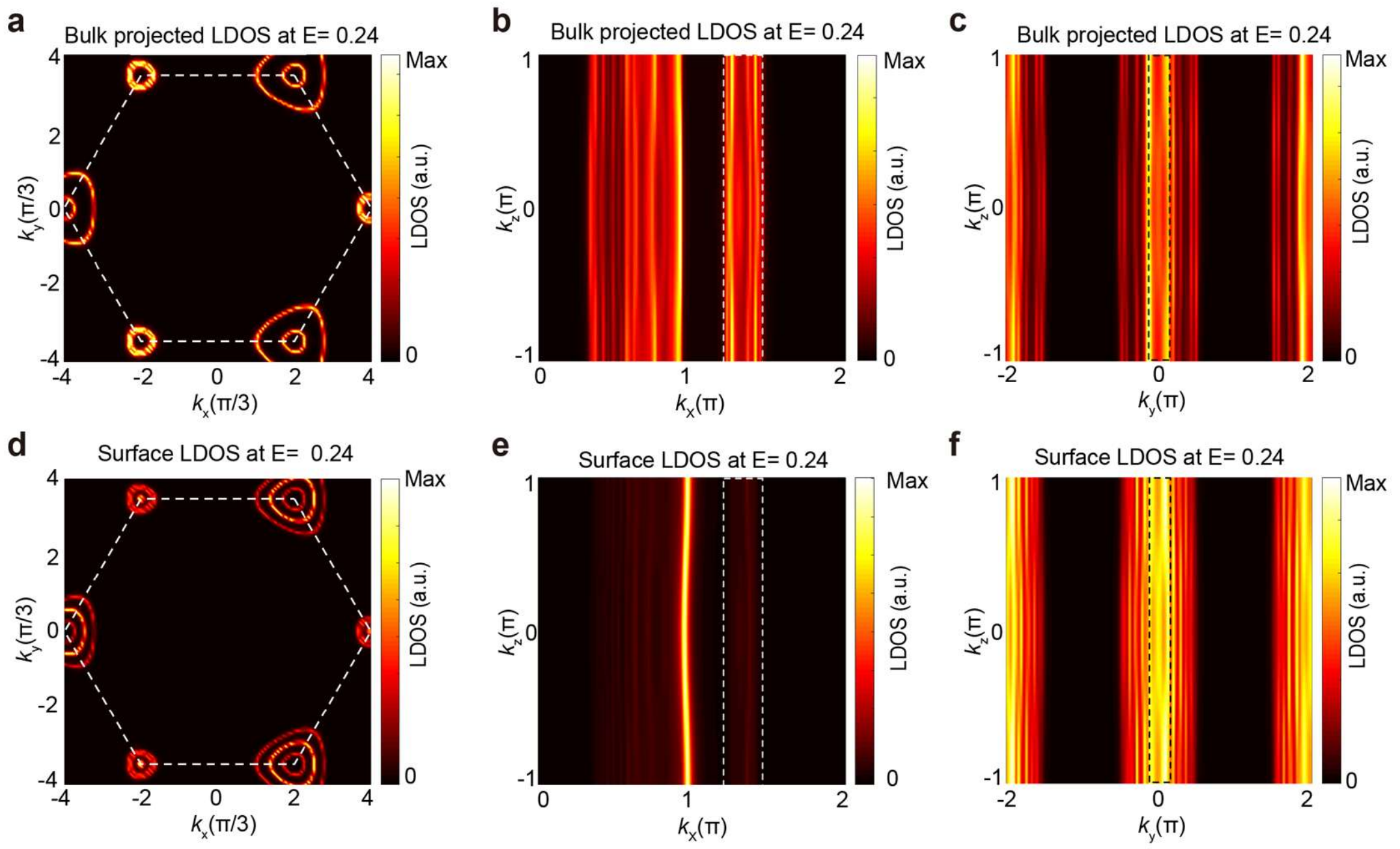

Fig. R9 | Theoretically calculated momentum-space LDOS at the nodal frequency E = 0.24. a-c Bulk isofrequency contours projected onto the k_x-k_y, k_x-k_z, and k_y-k_z planes, respectively. **d-f** Surface isofrequency contours on the k_x-k_y, k_x-k_z, and k_y-k_z planes, respectively.

To make this point clear, we have added the following discussions in the revised manuscript in lines 86-88 on page 3:

"The corresponding iso-frequency contours obtained from local-density-of-states (LDOS) calculations are presented in Supplementary Material, Sec. 2."

Figs. R8 and R9 have been added in Supplementary Materials as Figs. S1 and S2 with the following discussions:

"To verify the 3D nodal structures and avoid FFT resolution limits in finite-size 3D simulations, we employ the tight-binding Green's function method to calculate the momentum-resolved local density of states (LDOS). This approach generates the exact momentum-space spectral function $A(k, \mathrm{E})$, which serves as the theoretical equivalent of the isofrequency contours reconstructed from the measured near-field distributions.

To map the LDOS on different projection planes, we construct finite-thickness slab models along three lattice directions. For instance, to investigate the spectral properties projected onto the k_x-k_y plane, we construct an N_z-layer slab model $H_{slab}(k_\parallel)$ by opening the boundary along the *z*-direction while maintaining periodicity in the *x*-*y* plane. The retarded Green's function of the slab is defined as:

$$\mathrm{G}_{nn}(k_\parallel, \mathrm{E}) = \frac{1}{[(\mathrm{E} + \mathrm{i}\eta)\mathrm{I} - H_{slab}(k_\parallel)]} \tag{S3}$$

Where E is the targeted excitation energy, I is the identity matrix, and η is the intrinsic loss. The momentum-space LDOS for a specific layer *n* can then be extracted from the imaginary part of the partial trace of the Green's function:

$$A_n(k_\parallel, \mathrm{E}) = -\frac{1}{\pi} Im\left[\mathrm{Tr}\left(\mathrm{G}_{nn}(k_\parallel, \mathrm{E})\right)\right] \tag{S4}$$

By extracting the diagonal blocks corresponding to the bulk middle layer and the top surface layer, we can separately visualize the isofrequency contour projected onto the bulk and the surface.

Fig. S1 presents the theoretically calculated momentum-space LDOS at the nodal energy E = -0.24. At this specific energy, only the nodal structures around the *K*-*H* path (as depicted in Fig. 2c) are excited. As shown in the k_x-k_y projections (Fig. S1a and Fig. S1d), the white dashed hexagon delineates the boundary of the first Brillouin zone, around whose three vertices the nodal structure clearly emerges. Specifically, the nodal line projects onto the k_x-k_y plane as distinct singular points at these vertices, while the nodal surface manifests as bright continuous rings surrounding them. The geometric profile perfectly corroborates the 3D nodal structure illustrated in Fig. 2d.

In the k_x-k_z cross-sections (Fig. S1b and Fig. S1e), we indicate the positions of the nodal surfaces using dashed white lines. It is evident that the bulk spectra exhibit continuous vertical bright walls extending along the entire k_z direction around $k_x = 2\pi/3$. Furthermore, the antichiral surface states are prominently bright at this energy in Fig. S1e, which is in excellent agreement with the projected band dispersion depicted in Fig. 2e.

Finally, the k_y-k_z projections illustrated in Fig. S1c and Fig. S1f further confirm the phenomenon that the nodal structure extends continuously along the whole k_z direction around k_y =0, which is consistent with Fig. 2d. Similarly, the corresponding isofrequency contour at E = 0.24 is presented in Fig. S2, exhibiting similar topological features."

<u>Referee #2 -- Comment e:</u>

If possible, experimental verification of robustness would be preferable to numerical simulations

<u>Response from Authors:</u>

We sincerely thank Referee #2 for this valuable suggestion. We fully agree that direct experimental verification provides more compelling evidence for the topological robustness of the antichiral hinge states. To address this problem, we fabricated a new 3D gyromagnetic photonic crystal with sharp corners and measured the electric-field

distributions of the antichiral hinge states (as shown in Figs. R10a-R10c). Both the simulated and measured results clearly demonstrate that the antichiral hinge states propagate smoothly around the sharp corner without backscattering, thereby verifying their topological robustness. Moreover, we further introduced a lattice defect along the propagation path of the antichiral hinge states by removing a YIG rod together with its pair of permanent magnets, as shown in Figs. R10d-R10f. Both the simulated and measured electric-field profiles demonstrate that the hinge states circumvent the defect with negligible attenuation. Together, these results confirm the topologically protected robustness of the antichiral hinge states.

The Fig. R10 has been added as Fig. 5 in the revised manuscript with the following discussion:

"**Experimental demonstration of the robustness of antichiral hinge states**
Finally, we investigate the robustness of the antichiral hinge states. We first designed and fabricated a 3D gyromagnetic photonic crystal incorporating a sharp corner, as shown in Fig. 5a. Perfect-electric-conductor boundary conditions were imposed on two surfaces of the sample, whereas an absorbing boundary condition was applied to the third (see the experimental setup in Supplementary Material, Sec. 9). The electric-field distributions obtained from full-wave simulations (Fig. 5b) and microwave near-field measurements (Fig. 5c) show that the antichiral hinge states smoothly navigate the sharp corner without discernible backscattering. We further introduced a lattice defect along their propagation path by removing a YIG rod together with its pair of permanent magnets, as shown in Fig. 5d. Both the simulated (Fig. 5e) and measured (Fig. 5f) electric-field profiles demonstrate that the hinge states circumvent the defect with negligible attenuation. The measured transmission spectra and dispersions in the presence of the structural defect are provided in Supplementary Material, Sec. 10. Together, these results confirm the topologically protected robustness of the antichiral hinge states."

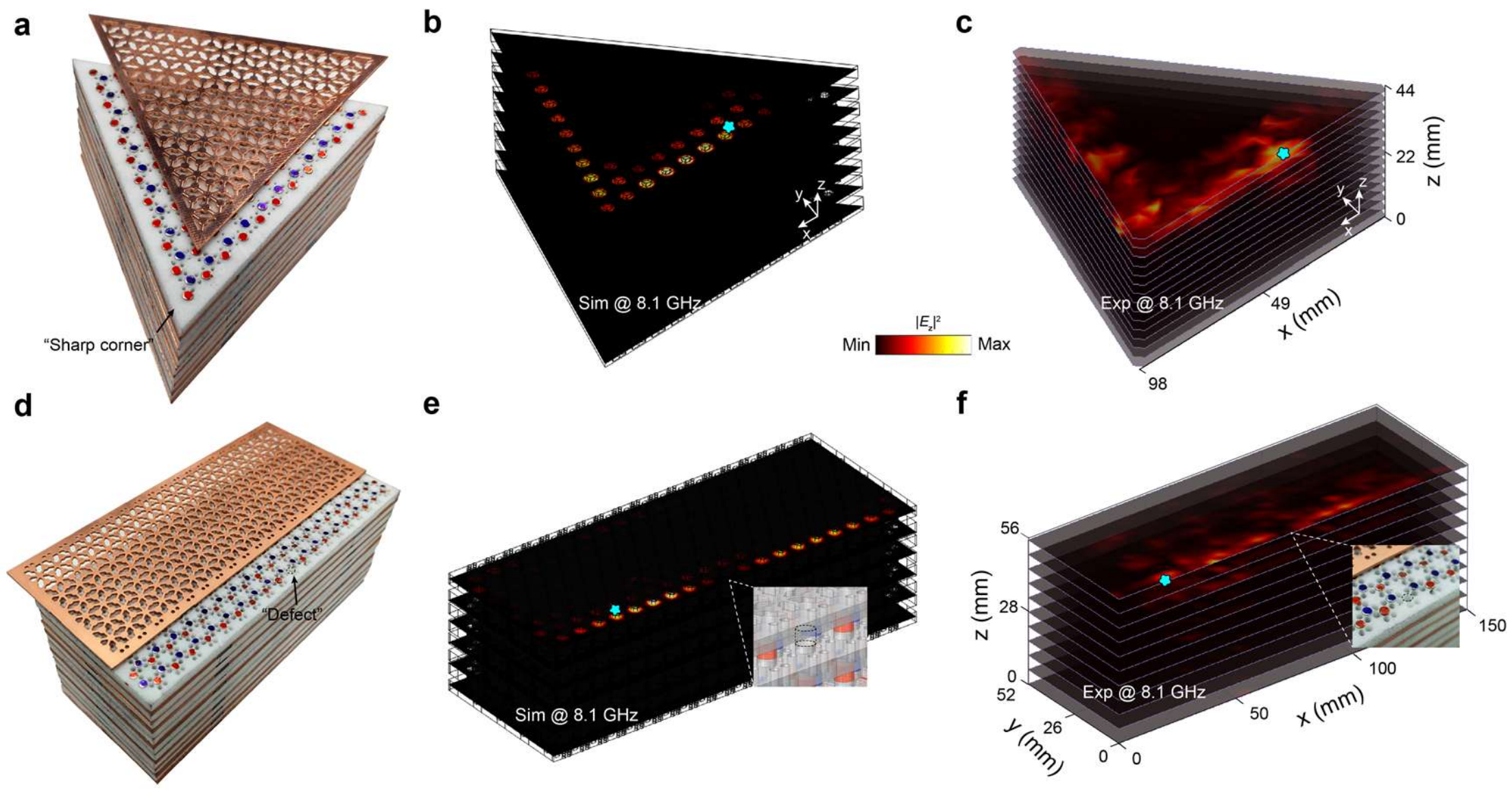

Fig. R10 | Experimental demonstration of the robustness of antichiral hinge states against sharp corners and structural defects. a Photograph of a fabricated 3D gyromagnetic photonic crystal with sharp corners. **b, c** Simulated (b) and measured (c) electric field distributions of the antichiral hinge states route around a sharp corner smoothly without backscattering. **d** Photograph of a fabricated 3D gyromagnetic photonic crystal containing a lattice defect in the top hinge. **e, f** Simulated (e) and measured (f) electric field distributions of the antichiral hinge states propagate through a lattice defect without backscattering.

GENERAL COMMENTS FROM REFEREE #3:

In this manuscript, Wang and coauthors experimentally demonstrate higher-order 1D antichiral hinge states in a 3D gyromagnetic photonic crystal. Its 3D structure appears to arise from a z-directional SSH-like stack of 2D antichiral semimetals. The emergence of kx hinge states is explained by the combined effects of nontrivial out-of-plane pz polarization and in-plane py polarization. Overall, the current work is novel and timely, and the results are correct and reasonable. I recommend publishing this manuscript in Nature Communications after the authors address the following issues.

Response from Authors:

We sincerely thank Referee #3 for the positive assessment of our manuscript and for recognizing our work as 'novel' and 'timely'. Below, we will address his/her specific comments point by point.

SPECIFIC COMMENTS FROM REFEREE #3:

Referee #3 -- Comment 1:

In the introduction, the authors describe chiral hinge states as "propagating nonreciprocally in opposite directions along two pairs of parallel hinges", citing Refs [20-22] about photonic axion insulators. However, this transport behavior differs from non-coplanar chiral hinge states characteristic of axion insulators, as the latter do not host parallel hinge transport on the surfaces. The chiral hinge states shown in Fig. 1(c) are more like those realized in the Chern-insulator stack with SSH modulation reported in PRB 111, L041112 (2025), rather than being directly related to axion cases.

Response from Authors:

We thank Referee #3 for pointing out this key distinction in transport behaviors of chiral hinge states. As correctly noted, the chiral hinge states in axion insulators are non-coplanar, whereas the parallel configuration in Fig. 1(c) corresponds to a Chern-insulator stack with SSH modulation.

To avoid confusion, we have added the suggested reference (Ref. [20]) in the revised introduction to accurately describe the origin of these parallel chiral hinge states.

Referee #3 -- Comment 2:

The theoretical analysis is based on a 3D tight-binding model with quite different parameter values: t1=1, t4=0.11, but t3~0.01. Such a choice leads to an extremely weak interlayer coupling, which can also be seen in Fig. 2c and Fig. 3b with flat bands along the KH, K'H', and gamma-A directions. Thus, one may doubt the topological properties remain unchanged even when t3=0. Then, the difference between antichiral hinges in 3D and antichiral edges in 2D seems marginal.

Response from Authors:

We sincerely thank Referee #3 for this insightful question. We fully understand the Referee's concern that small interlayer coupling ($t_3 \approx 0.01$) leads to nearly flat bands along k_z-direction.

However, we would like to clarify that the magnitude of t_3 is not the key factor for the 3D topological phase. Rather, it is the contrast between the interlayer couplings t_3 and t_4 that drives the topological phase transition. Choosing a small t_3 here is primarily

for simplicity in the photonic design, as it allows us to focus on the essential physics without loss of generality.

To address the Referee's concern, we have performed calculations with larger interlayer couplings ($t_3 = 0.1, t_4 = 0.5$). As depicted in Fig. R11a, the band structure preserves similar physical properties, featuring the coexistence of the nodal surface (NS, cyan sphere) and nodal line (NL, purple line) semimetal phases. Due to the larger interlayer couplings, the bands along the *K*-*H*, *K'*-*H'*, and Γ-A directions now exhibit tilted dispersions. The corresponding visualization of these NSs and NLs in 3D momentum space is shown in Fig. R11b, exhibiting a similar NS-wrapped NL configuration as that in Fig. 2d. Furthermore, the projected band dispersion is presented in Fig. R11c, where the tilted, four-fold-degenerate antichiral hinge states (red line) precisely connect the projections of the NSs (cyan regions).

Finally, as shown in Fig. R11d, applying the nested Wilson loop method to the full 3D Hamiltonian yields a strictly quantized nested polarization $p_y^{v_z}$ of 1/2 exactly within the momentum region $k_{s1}<k_x<k_{s2}$, defined by the projected NSs. This non-trivial quantization unambiguously demonstrates that the antichiral hinge states are globally protected by the 3D bulk topology, completely distinguishing them from the antichiral edge states in 2D systems.

The complete theoretical derivations and the calculation of the nested polarization $p_y^{v_z}$ are already provided in our response to Referee #2 (Comment 3). To avoid redundancy, we respectfully refer the Referee #3 to that discussion for further details.

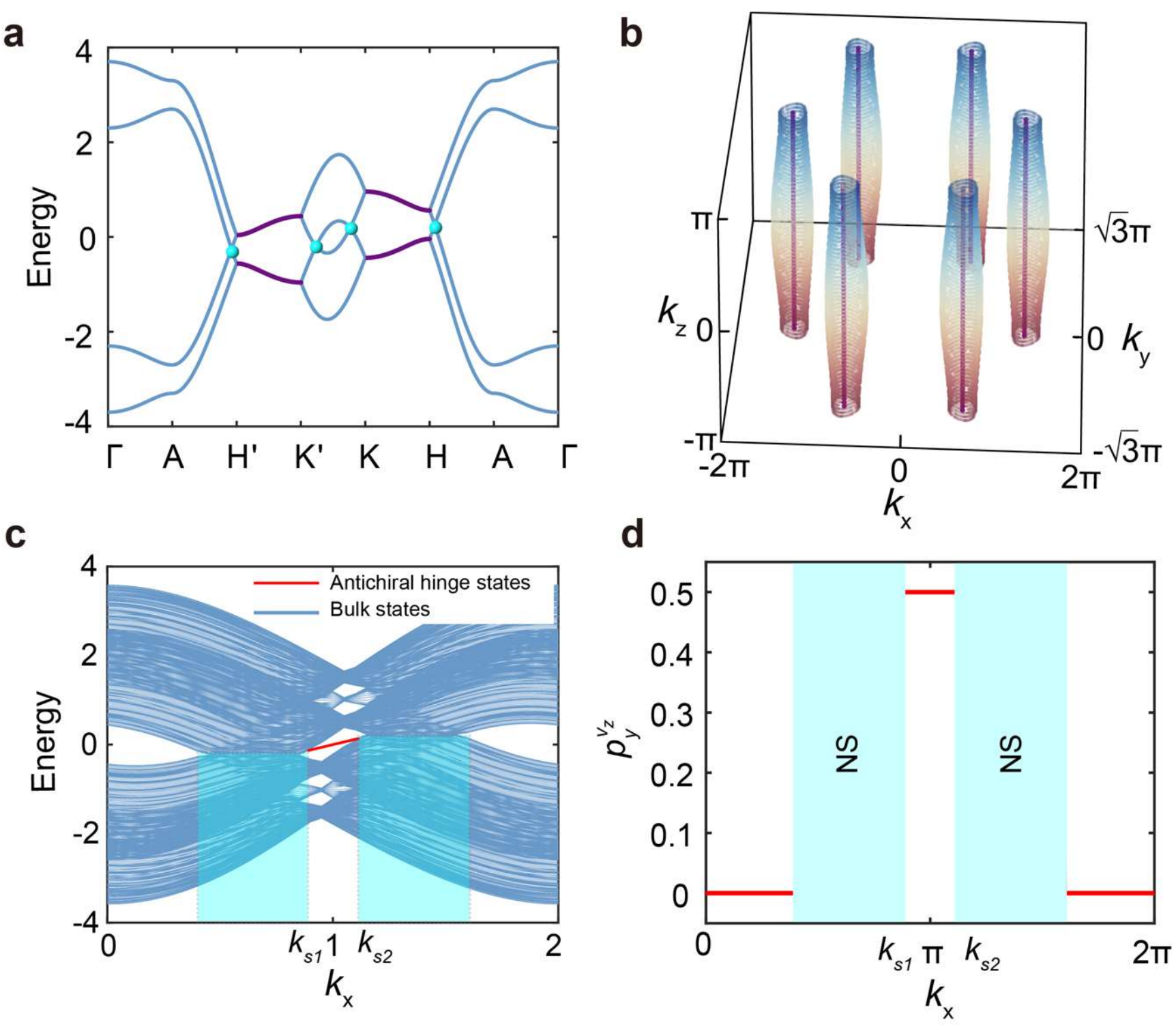

Fig. R11 | Theoretical model with larger interlayer couplings. a Calculated bulk band structure of the 3D tight-binding model with parameters M=0, t_1=1, t_2=0.05, t_3=0.1, t_4=0.5, $\varphi = \frac{\pi}{2}$. **b** Visualization of the nodal surfaces (colored surfaces) and

nodal lines (purple lines) in the 3D momentum space. **c** Projected band dispersions of a 3D modified Haldane model with dimerized interlayer coupling that is finite in the y- and z-directions and periodic in the x-direction. **d** Nested polarization calculated from the full 3D Hamiltonian.

We also calculate the band dispersion of the antichiral hinge states (red dots) with small and larger interlayer couplings, as shown in Fig. R12, from which we can see that the interlayer couplings have almost no influence on the antichiral hinge states.

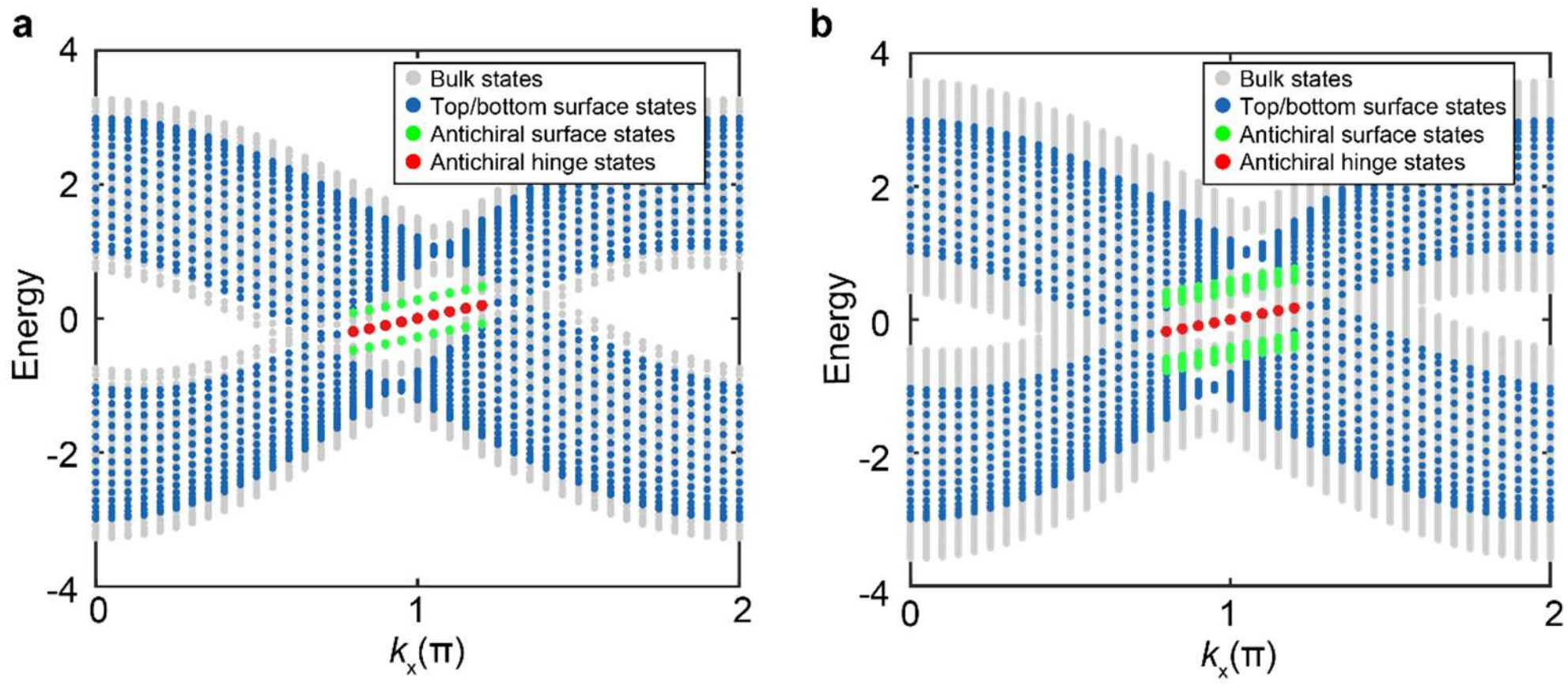

Fig. R12 | Detailed projected dispersion along the k_x direction with small and larger interlayer couplings. **a** Dispersion computed with small interlayer couplings (M = 0, t_1 = 1, t_2 = 1/18, φ = π/2, t_3 = 0.2t_2, and t_4 = 5t_2). **b** Dispersion with larger interlayer couplings (t_3 = 0.1, t_4 = 0.5), corresponding to Fig. S5. The red and green circles highlight the antichiral hinge and surface states, respectively. The gray and blue dots indicate the bulk and top- and bottom-surface states.

To make this point clear, we have added the following discussions to the revised Supplementary Materials:

"In the main text, we chose relatively weak interlayer couplings to facilitate the practical design and fabrication of the experimental sample. This choice of parameter, however, does not alter the underlying topological phase. To demonstrate this explicitly, we calculated the band structures for stronger interlayer couplings. As shown in Fig. S5a, the system retains the same topological characteristics, including the robust coexistence of nodal-surface (NS) and nodal-line (NL) semimetal phases. Owing to the enhanced interlayer couplings, the bands along the *K–H, K′–H′*, and *Γ–A* directions acquire pronounced tilted dispersions. The corresponding NSs and NLs in 3D momentum space are visualized in Fig. S5b. The projected band structure in Fig. S5c further shows tilted, fourfold-degenerate antichiral hinge states that connect the projected NSs. Finally, as shown in Fig. S5d, the nested Wilson-loop calculation for the full 3D Hamiltonian yields a quantized nested polarization, $p_y^{(v_z)} = 1/2$, throughout the momentum interval $k_{s1} < k_x < k_{s2}$ defined by the projected NSs. This nontrivial quantization demonstrates that the antichiral hinge states in 3D systems are globally protected by the 3D bulk topology, distinguishing them fundamentally from the antichiral edge states in 2D systems."

Fig. R11 has been adopted as Fig. S5 in the revised Supplementary Materials.

Fig. R12 has been adopted as Fig. S3 in the revised Supplementary Materials.

Referee #3 -- Comment 3:
The measured transmission in Fig. 4(b-c) shows relatively high values over 7-9 GHz except the hinge state window. However, the measured dispersions in Fig. 4(f-g) are unexpectedly clean, with nearly no background states aside from the narrow hinge state window (7.9-8.3 GHz). It is unlike previously semimetal experiments (Refs. [15-17]). The authors would like to clarify this point.

Response from Authors:
We thank Referee #3 for raising this point. We agree that the elevated background in the original Figs. 4b and 4c appeared inconsistent with the clean dispersions shown in Figs. 4f and 4g.

This discrepancy arose because the transmission measurements and near-field scans were performed at different stages of the experiment. The near-field scans were acquired later using an optimized excitation configuration, including improved antenna coupling and source positioning. This configuration suppresses bulk scattering and preferentially excites the antichiral hinge states.

To ensure consistency, we have re-measured the transmission spectra using the same optimized configuration. As shown in the Figs. R13 and R14, the updated transmission spectra exhibit substantially reduced bulk background and are now consistent with the clean hinge-state dispersions.

In comparison with previous semimetal experiments (Refs. [15–17]), bulk and hinge states were simultaneously excited in those studies. We likewise observed mixed bulk–hinge dispersion features under certain excitation conditions. However, by adjusting the source position, we can selectively couple to the hinge states while strongly suppressing bulk contributions, thereby obtaining the clean dispersions presented here.

Similar clean hinge-state dispersions have also been reported in recent studies [Nat. Commun. 14, 6636 (2023); Phys. Rev. Lett. 132, 186601 (2024); Phys. Rev. Lett. 135, 056601 (2025)].

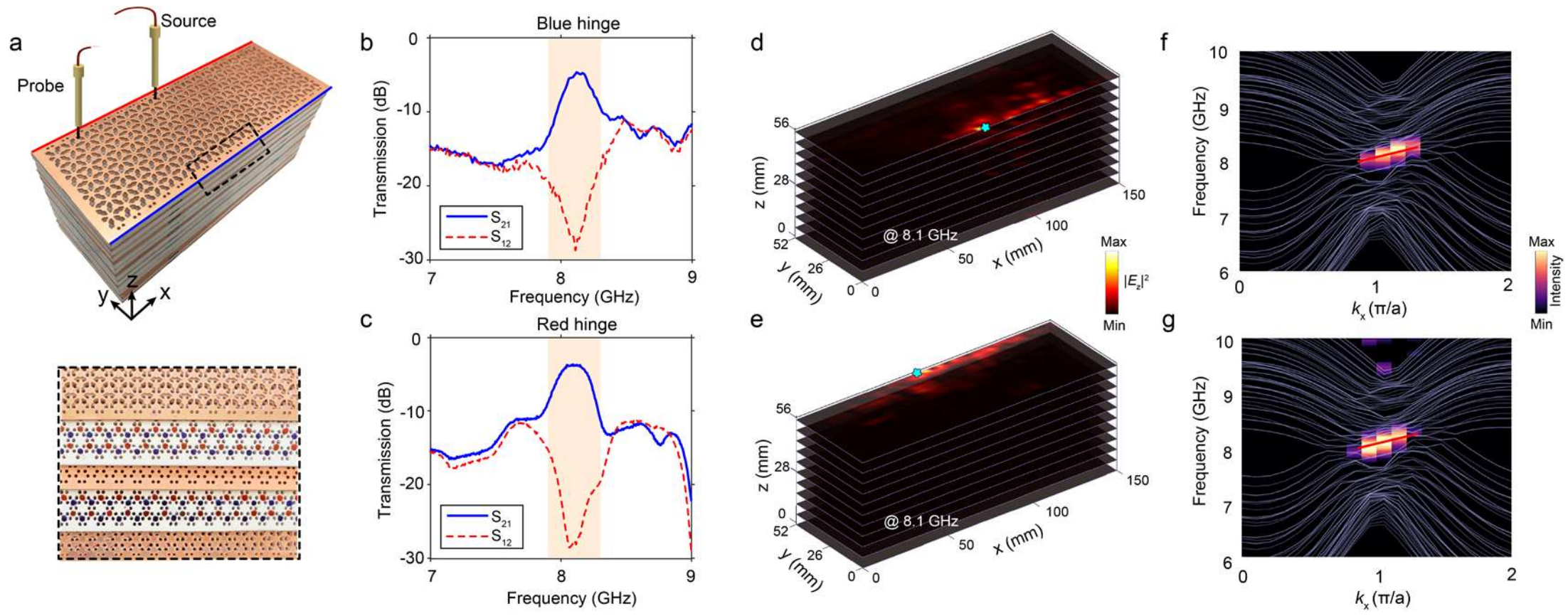

Fig. R13 | Experimental observation of antichiral hinge states. a Upper panel: Photograph of the fabricated 3D gyromagnetic photonic crystal that consists of $20 \times 4 \times 5$ unit cells in the x, y, and z directions, respectively. Lower panel: Enlarged photographs of the perforated copper plates and air foams (white), respectively. Red and blue disks denote the magnetized directions of gyromagnetic rods at different sublattice sites. **b, c** Measured forward (S_{21}, blue line) and backward (S_{12}, red line) transmission spectra at the two top hinges. The orange-shaded regions indicate the frequency range of the antichiral hinge states. **d, e** Measured electric field distributions of the antichiral hinge states at 8.1 GHz, with the source antenna (cyan star) positioned at the center of the hinges. **f, g** Measured (colormaps) and simulated (red lines) dispersions of the antichiral hinge states at the two top hinges along the k_x direction.

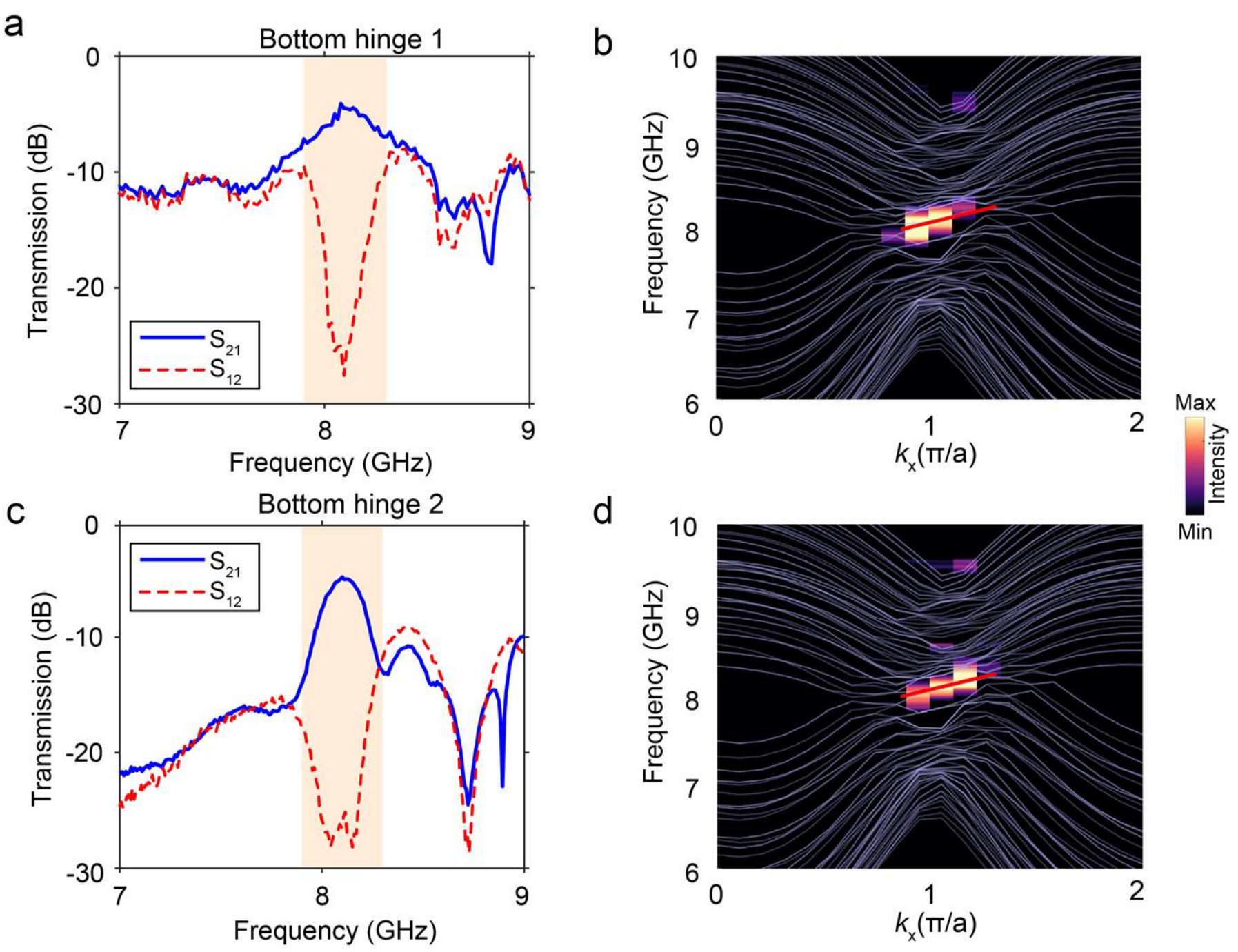

Fig. R14 | Measured transmission spectra and dispersions of antichiral hinge states at two bottom hinges. a, **c** Measured forward (S_{21}) and backward (S_{12}) transmission spectra of the antichiral hinge states at the two bottom hinges. **b**, **d** Measured (colormaps) and simulated (red lines) dispersions of the antichiral hinge states at the

two bottom hinges along the k_x direction.

Fig. R13 has been adopted as Fig. 4 in the revised manuscript.

Fig. R14 has been adopted as Fig. S7 in the revised Supplementary Materials.

Referee #3 -- Comment 4:
The red/blue color lines in Fig. 4 are somewhat confusing and inconsistent. The authors may consider using dashed lines to plot S12 parameters.

Response from Authors:
We thank Referee #3 for this helpful suggestion. We fully agree that using dashed lines enhances the readability of the transmission spectra. Following your advice, we have updated the corresponding figures in both the revised main text and the Supplemental Materials.

Referee #3 -- Comment 5:
In Supplemental Material Fig. S1, severe overlap of data hinders readability. And, it would be better if the authors could explain how to technically distinguish the overlapping bulk, surface, and hinge states.

Response from Authors:
We thank Referee #3 for this constructive comment. To eliminate visual ambiguity, we have replotted the dispersion spectra by classifying the eigenmodes based on their spatial field distributions, as shown in Fig. R15.

To make this clearer, we have added the following discussion to the revised Supplementary Materials:

"In our numerical calculations, we evaluate the spatial energy distribution of each eigenmode within the 3D supercell. We partition the lattice sites into four regions: the bulk interior, the top and bottom facets, the side facets, and the 1D hinges at the intersections of adjacent side facets. By integrating the field intensity over each region, we classify each mode as a bulk state, a top/bottom surface state, an antichiral side-surface state, or a hinge state according to the region where its energy is predominantly localized. We present detailed projected dispersions for the parameters used in the main text (Fig. S3a) and for a case with stronger interlayer coupling along the *z*-direction (Fig. S3b), corresponding to Fig. S5."

Fig. R15 has been included as Fig. S3a in the revised Supplementary Materials.

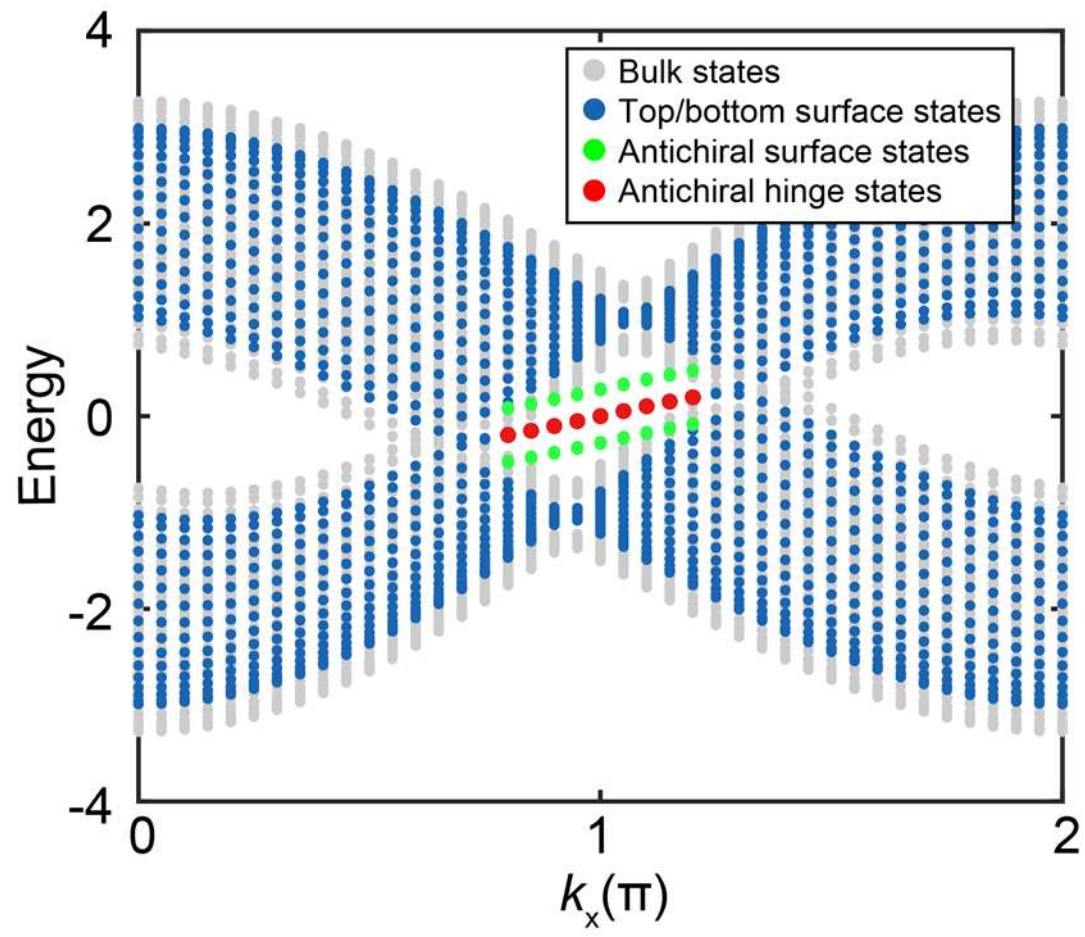

Fig. R15. Detailed projected dispersion along the k_x direction. The red and green circles highlight the antichiral hinge and surface states, respectively. The grey and blue circles indicate the bulk states and the top and bottom surface states, respectively.

Response Letter to Referees

We sincerely thank the three Reviewers for their careful reassessment of our revised manuscript (NCOMMS-26-026679B). We are grateful that Reviewers #1 and #3 recommend publication and that Reviewer #2 considers the manuscript publishable in principle. In the text below, the referee's comments are quoted in *italics* and are followed by the corresponding detailed response. We also revised the manuscript and the supplementary materials based on the referee's comments and suggestions, and these updates are highlighted in blue in those files. In the text below, references to these updates are also highlighted.

GENERAL COMMENTS FROM REFEREE #1:

The authors have provided thorough and satisfactory responses to all my queries. I am therefore happy to recommend this manuscript for publication in Nature Communications.

Response from Authors:

We sincerely thank Reviewer #1 for the positive assessment of our revision and for recommending the manuscript for publication. We greatly appreciate the Reviewer's constructive comments throughout the review process, which have helped us improve the manuscript's clarity and rigor.

GENERAL COMMENTS FROM REFEREE #2:

Overall, I think the authors have addressed most of my concerns in the revised manuscript. I can recommend its publication in principle. However, I would like to raise one remaining issue regarding my previous Comment 3.

The hinge states discussed in this work are extrinsic, meaning that they can be removed by adding additional surfaces or hinges. Consequently, their topology is determined by the topological properties of the 2D surface states rather than by an intrinsic 3D bulk topological invariant. This is consistent with the well-established concept of surface-obstructed topology. Therefore, I believe the statement in the manuscript that the hinge modes are "protected by the 3D band topology" is somewhat misleading and should be revised. A more accurate description would clarify that the hinge states originate from the topology of the gapped surfaces (or the surface-obstructed topology) rather than from an intrinsic higher-order bulk topological phase.

Response from Authors:

We thank Reviewer #2 for identifying this important distinction, and we fully agree. Our previous wording conflated two different aspects of the system: (i) the three-dimensional bulk semimetallic band structure, characterized by coexisting nodal lines and nodal surfaces, and (ii) the termination-dependent topology of the gapped surfaces that gives rise to the hinge states. The bulk nodal structure organizes the projected spectrum and the momentum interval in which the boundary bands occur, and it supplies the gapless compensation required for antichiral propagation. It does not, however, supply an intrinsic three-dimensional higher-order bulk invariant that enforces the hinge modes independently of boundary termination.

To rigorously demonstrate this and directly address the Reviewer's physical insight, we have explicitly calculated the surface Wannier polarization $v_z^{\text{surface}}(k_x)$ for the y-normal surfaces. By isolating the antichiral surface band and tracking its evolution along k_z, our numerical results confirm that v_z^{surface} is strictly quantized to 0.5 exactly within the momentum interval 2/3 < k_x(π) < 4/3. The nontrivial quantization within this specific domain provides mathematical evidence that the 1D antichiral hinge channels are a manifestation of boundary-obstructed topology.

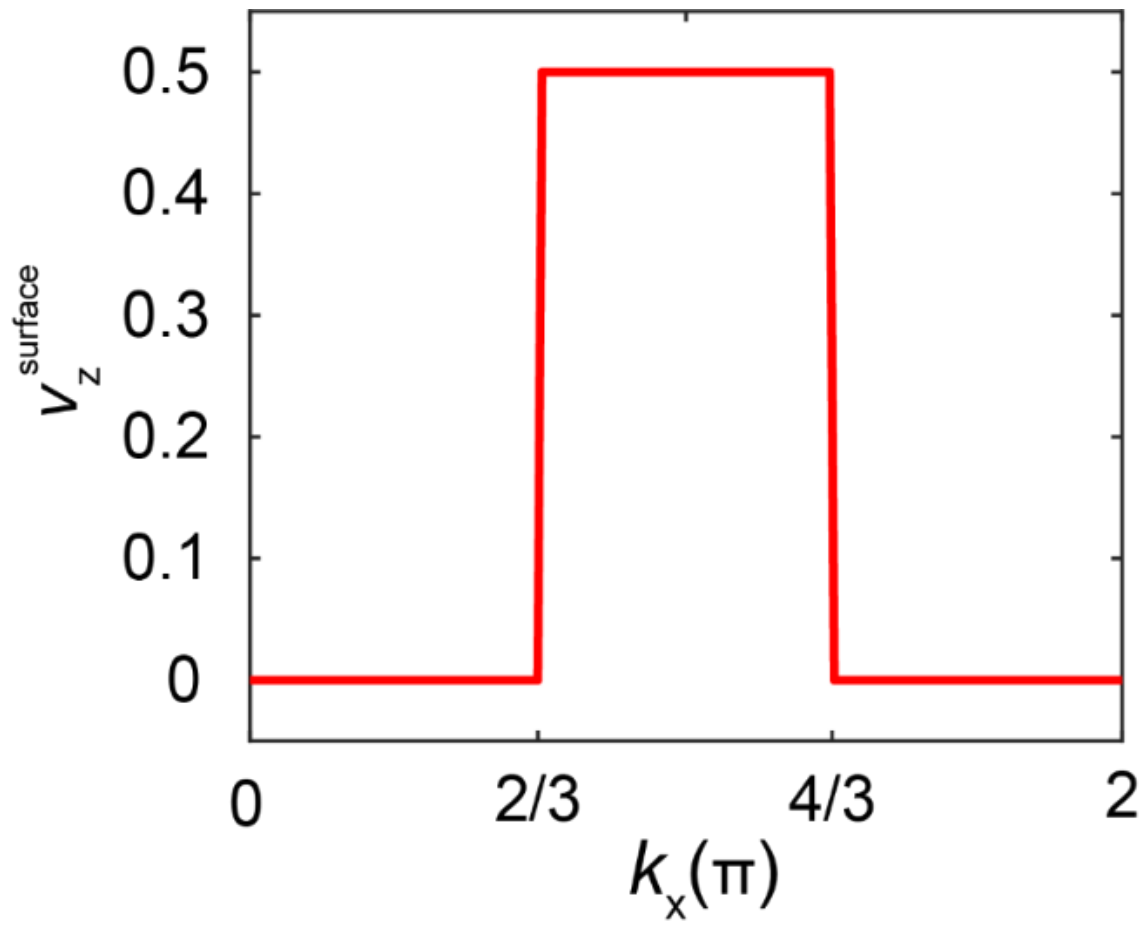

Fig. R1 | Surface Wannier polarization of the *y*-normal surface. Calculated surface polarization v_z^{surface} as a function of the wavevector k_x.

The description of the topological invariant in the main text has been revised as follows:

"To establish the topological origin of the antichiral hinge states [40], we use the nested Wilson-loop method to calculate the nested polarization $p_y^{v_z}$ throughout the 3D momentum space (see Supplementary Material, Sec. 5 for the detailed calculation). As shown in Fig. 2f, $p_y^{v_z}$ is quantized to $1/2$ within the momentum interval bounded by the inner projected boundaries of the NSs. This half-quantized polarization is a hallmark of higher-order topology. Notably, the nontrivial momentum region identified by this quantized 3D invariant coincides precisely with the momentum interval over which the antichiral hinge states appear. In our 3D SSH-stacked architecture, the emergence of the antichiral hinge states depends on the specific surface termination, which is a hallmark of surface-obstructed topology [41-42]. To rigorously demonstrate this, in addition to the nested Wilson loop evaluated from the full 3D Brillouin zone, we further calculate the surface polarization [see Supplementary Material, Sec. 5 for the detailed calculation]. The non-trivial quantization of this surface polarization provides direct mathematical evidence that the hinge states are protected by the topology of the gapped surfaces. (see Supplementary Material, Sec. 6 for the theoretical calculation with larger out-of-plane couplings)."

The following description has been added to Section 5 of the Supplementary Material:

"To explicitly confirm that the antichiral hinge states originate from surface-obstructed topology, we evaluate the surface Wannier polarization $v_z^{\text{surface}}(k_x)$ for the y-normal

surfaces. While the nested Wilson loop captures the global 3D bulk properties, our antichiral hinge states emerge directly from the gapped 2D surface spectrum. By isolating the antichiral surface band and tracking its evolution along k_z, we compute the one-dimensional Zak phase:

$$v_z^{\text{surface}}(k_x) = \frac{1}{2\pi}\oint i\langle\psi^{\text{surface}}(k_x, k_z)|\partial_{k_z}|\psi^{\text{surface}}(k_x, k_z)\rangle\, dk_z \pmod 1 \quad \text{(S9)}$$

As shown in Fig.S5, the numerical results reveal v_z^{surface} that is strictly quantized to 0.5 exclusively within the momentum interval 2/3 < $k_x(\pi)$ < 4/3. The nontrivial quantization within this specific domain provides mathematical evidence that the 1D antichiral hinge channels are a manifestation of boundary-obstructed topology."

The Fig. R1 has been added to the revised Supplementary Materials as Fig. S5.

GENERAL COMMENTS FROM REFEREE #3:

The authors have well addressed my concerns arising in the first round of review. I recommend its publication in Nature Communications.

Response from Authors:

We sincerely thank Reviewer #3 for the positive assessment of our revision and for recommending the manuscript for publication. We are grateful for the Reviewer's constructive suggestions, which have helped us strengthen the manuscript.
